# Effective Oncoleaking Treatment of Pancreatic Cancer by Claudin-Targeted Suicide Gene Therapy with *Clostridium perfringens* Enterotoxin (CPE)

**DOI:** 10.3390/cancers13174393

**Published:** 2021-08-31

**Authors:** Jessica Pahle, Dennis Kobelt, Jutta Aumann, Diana Behrens, Ole Daberkow, Margarita Mokritzkij, Jörg Piontek, Ulrike Stein, Wolfgang Walther

**Affiliations:** 1 Experimental and Clinical Research Center, Charitè-Universitätsmedizin Berlin and Max-Delbrück-Center for Molecular Medicine, Robert-Rössle-Str. 10, 13125 Berlin, Germany; jessica.werchan@gmail.com (J.P.); dennis.kobelt@epo-berlin.com (D.K.); jutta.aumann@mdc-berlin.de (J.A.); Margarita.mokritzkij@mdc-berlin.de (M.M.); ustein@mdc-berlin.de (U.S.); 2 German Cancer Consortium, Deutsches Krebsforschungzentrum (DKFZ), 69120 Heidelberg, Germany; 3 Experimental Pharmacology & Oncology (EPO) GmbH Berlin-Buch, Robert-Rössle-Str. 10, 13125 Berlin, Germany; diana.behrens@epo-berlin.com (D.B.); ole.daberkow@epo-berlin.com (O.D.); 4 Nutritional Medicine, Medical Department, Division of Gastroenterology, Infectiology, Rheumatology, Institute of Clinical Physiology, Charitè-Universitätsmedizin Berlin, 12203 Berlin, Germany; joerg.piontek@charite.de

**Keywords:** *Clostridium perfringens* enterotoxin (CPE), pancreatic cancer, gene therapy, suicide gene, combination therapy

## Abstract

**Simple Summary:**

Current therapies for pancreas carcinoma (PC) are of limited efficacy due to tumor aggressiveness and therapy resistance. Bacterial toxins with pore-forming (oncoleaking) potential are promising tools in cancer therapy. We have developed a novel, suicide gene therapy treatment, based on *Clostridium perfringens* enterotoxin (CPE)-mediated oncoleaking. This is achieved by CPE suicide gene therapy to treat PC, which overexpresses the claudin-3 and -4 (Cldn3/4) tight junction proteins, which are targets of CPE action. This targeted gene therapy causes rapid eradication of Cldn3/4 overexpressing PC cells via oncoleaking and initiation of apoptotic/necrotic signaling. We demonstrate efficacy of this approach in vitro and after nonviral in vivo gene transfer in cell lines and in patient derived xenograft PC models. This therapy approach has translational potential for treatment of pancreas carcinomas and could also be translated into new combination settings with conventional chemotherapy.

**Abstract:**

Pancreatic cancer (PC) is one of the most lethal cancers worldwide, associated with poor prognosis and restricted therapeutic options. *Clostridium perfringens* enterotoxin (CPE), is a pore-forming (oncoleaking) toxin, which binds to claudin-3 and -4 (Cldn3/4) causing selective cytotoxicity. Cldn3/4 are highly upregulated in PC and represent an effective target for oncoleaking therapy. We utilized a translation-optimized CPE vector (optCPE) for new suicide approach of PC in vitro and in cell lines (CDX) and patient-derived pancreatic cancer xenografts (PDX) in vivo. The study demonstrates selective toxicity in Cldn3/4 overexpressing PC cells by optCPE gene transfer, mediated by pore formation, activation of apoptotic/necrotic signaling in vitro, induction of necrosis and of bystander tumor cell killing in vivo. The optCPE non-viral intratumoral in vivo jet-injection gene therapy shows targeted antitumoral efficacy in different CDX and PDX PC models, leading to reduced tumor viability and induction of tumor necrosis, which is further enhanced if combined with chemotherapy. This selective oncoleaking suicide gene therapy improves therapeutic efficacy in pancreas carcinoma and will be of value for better local control, particularly of unresectable or therapy refractory PC.

## 1. Introduction

Pancreatic cancer (PC) is one of the leading causes of cancer death in developed countries and one of the most lethal malignancies worldwide [1,2]. PC has one of worst prognosis, reflected by a 5-year survival rate of less than 5% and median survival of less than 6 months [3,4]. Despite advances in molecular and targeted therapies for improved patient survival of many different tumor entities, the outcome for PC has not changed much over the past 30 years. Monotherapy with gemcitabine is still the standard of care therapy for PC [5], as no combination treatment—neither with 5-fluorouracil (5-FU), cisplatin nor carboplatin—showed a significant increase of overall survival [6,7]. By contrast, improvements for increased median overall survival were made by the 5-FU-based triplet chemotherapy FOLFIRINOX (oxaliplatin, irinotecan, 5-FU and leucovorin) [8]. The only potentially curative treatment of pancreatic cancer is surgical resection, which can lead to significantly longer survival compared to other treatment modalities [9].

Apart from all the established treatment modalities, gene therapy might be an alternative for an effective PC therapy. In particular, suicide gene therapy with use of bacterial toxins is attractive. Bacterial toxins are produced and released by bacteria affecting different targets in host cells. An increasing number of in vitro and in vivo studies using different bacterial toxins demonstrate efficacy in cancer cell killing [10]. During the last years, improved processing and manipulation of bacterial toxins, such as diphtheria toxin, streptolysin O or *Clostridium perfringens* enterotoxin (CPE) as well as their encoding gene sequences translated into toxin-based cancer therapy [11,12,13,14].

CPE is of particular interest, since it allows effective and targeted attack of cancer cells. The toxin is produced by the Gram-positive, anaerobic bacterium *Clostridium perfringens* [15]. This protein of 35 kDa comprises 319 amino acids. Its C-terminal receptor-binding domain (cCPE, residues 194 to 319) recognizes and binds to the tight junction proteins claudin-3 and -4 (Cldn3/4). The N-terminal region of CPE is involved in oligomerization and pore formation [16,17] and the cCPE domain mediates high-affinity binding to claudins. Pore formation, however, is exclusively initiated by the N-terminal residues 80–160, known as TM1 region [12,18,19,20,21]. High CPE concentration causes massive pore formation, which leads to rapid Ca^2+^ influx and consequently to necrotic cell death. Low CPE concentration, however, results in low pore numbers, rather leading to apoptotic cell death [21,22].

Numerous reports showed that compared to normal tissues, particularly epithelial cancers, such as pancreatic, colon, ovarian, breast and prostate cancer possess high Cldn3/4 expression and dysregulation [23,24,25]. Based on this fact and regarding the cytotoxic efficacy of CPE, considerable efforts were made to develop a CPE-based targeted cancer therapy for Cldn3/4 overexpressing tumors. Numerous studies demonstrated that in vitro and in vivo treatment with recombinant CPE exerted cytotoxic effects via pore formation (oncoleaking) on high Cldn3/4 expressing pancreatic, ovarian and breast cancer cells, which was associated with tumor reduction or elimination [26,27,28]. Since binding of CPE to claudins is highly specific, this toxin is attributed with great potential for targeted gene therapeutic treatment of Cldn3/4 overexpressing tumors [29,30]. Based on this, we exploited CPE as a valuable option for therapy refractory tumors such as pancreas carcinoma. In this study we used the toxin in a suicide gene therapy approach for efficient and targeted eradication of Cldn3/4 overexpressing PC as a novel oncoleaking therapy in vitro and more importantly in vivo.

## 2. Materials and Methods

For comprehensive and detailed description of all methods see Supplementary Methods.

### 2.1. Cell Lines

Human pancreas carcinoma AsP-1, BxPC-3, Capan-1 and Sk-Mel-5 melanoma cells were grown in RPMI medium (Gibco, Life technologies, Darmstadt, Germany), 10% FCS (Biochrom, Berlin, Germany). The human pancreas carcinoma HUP-T3, MIA PaCa-2, PA-TU-8902 and human colon carcinoma HT-29 cells were grown in DMEM (Gibco), 10% FCS (Biochrom). All lines were kept at 37 °C, 5% CO_2_. The identity of all cell lines was confirmed by STR-genotyping (DSMZ, Braunschweig, Germany).

### 2.2. Pancreatic Cancer Patient Derived Xenograft (PDX) Models

For in vivo studies, 21 pancreas carcinoma PDX models of stage IV (EPO Berlin-Buch GmbH, Berlin, Germany), were used [31].

### 2.3. Quantitative Real-Time RT-PCR

Total RNA was isolated using GeneMatrix Universal RNA Purification Kit EURx (Roboklon, Berlin, Germany) and reverse transcribed. Quantitative real-time PCR (qPCR) was performed with SYBR GREEN in the LightCycler 480 (Roche Diagnostics, Mannheim, Germany). They following primers were used for claudin-3: forward 5′-CTGCTCTGCTGCTCGTGTCC-3′; reverse 5′-TTAGACGTAGTCCTTGCGGTCGTAG-3′; for claudin-4: forward 5′-CCTCTGCCAGACCCATATAA-3′; reverse 5′-CACCGTGAGTCAGGAGATAA-3′; for RNA polymerase II: forward 5′-GCACCACGTCCAATGACA T-3′, reverse 5′-GTGCGGCTGCTTCCATAA-3′. Normalization was done with the housekeeping gene glucose-6-phosphate dehydrogenase (G6PDH) using the hG6PDH Roche Kit (Roche Diagnostics). Primers used for G6PDH: forward 5′-GAAGATGGTGATGGGATTTC-3′, reverse 5′-GAAGGTGAAGGTCGGAGT-3′.

### 2.4. Western Blot

Cells or tissue cryosections were lysed in RIPA buffer (50 mM TRIS, 150 mM, NaCl, 1% Nonidet P-40, 0.5% sodiumdeoxycholate, protease inhibitor, ddH_2_O). For isolation of membrane, cytosolic and nuclear fractions the Cell Fractionation kit (Cell Signaling, Frankfurt, Germany) was used. Lysates were electrophoresed in 10% precast NuPAGE gels (Invitrogen, Waltham, MA, USA) and transferred to nitrocellulose membranes. Membranes were blocked for 1 h at room temperature in TBS (50 mM Tris, 150 mM NaCl, pH 7.5, 5% fat-free dry milk and 2.5% casein) and washed in TBST (0.05% Tween 20 in PBS). Western blot was performed with primary rabbit anti-claudin-3 antibody (Acris, Origene Technologies, Rockville, MD, USA), rabbit anti-claudin-4 antibody (Acris), rabbit polyclonal anti-CPE (BioRad, Hercules, CA, USA), mouse monoclonal anti-β-tubulin (BD Bioscience, East Rutherford, NJ, USA) or mouse monoclonal anti-β-actin antibody (Pierce, Thermo Fisher Scientific, Waltham, MA, USA). As secondary HRP-labeled goat anti-rabbit-IgG antibody (Promega, Madison, WI, USA), HRP-labeled goat anti-mouse-IgG antibody (Pierce,) or goat anti-mouse IgM/IgG (Sigma-Aldrich, Merck, St. Louis, MI, USA) was added. Detection was done using ECL solution (Amersham, GE Healthcare, Chicago, IL, USA) and exposure to Kodak X-Omat AR film (Kodak, Stuttgart, Germany).

### 2.5. Clostridium Perfringens Enterotoxin (CPE) Expressing Plasmids

For transfection experiments the translation optimized pCpG-optCPE (optCPE) and the pcDNA3-CPE-GFP (CPE-GFP) plasmids were used [29,30]. Preparation of plasmid-DNA was done by Jetstar Plasmid Purification Maxi Kit (Genomed, Löhre, Germany).

### 2.6. Transfection of Cells with Plasmid DNA and siRNA

For transfection experiments, the pCpG-optCPE (optCPE) or the pCpG-mcsG2 empty vector control (VC) plasmid (Invivogen, San Diego, CA, USA) was used. A mutated optCPE construct unable to bind Cldn3/4 (mutCPE, CPE-Y306A/L315A; Figure S1) was used for in vivo selectivity testing [20,21,32,33,34]. For transfections, 3 × 10^5^–5 × 10^5^ cells/well were seeded in 6-well plates and after 24 h transfected with respective plasmid DNA plus transfection reagent (FuGene, X-treme, Metafectene, TransIT) according to manufacturer’s instruction. After 12–72 h, cells were washed with PBS and RNA or protein was isolated.

Knockdown of Cldn3/4 was done with pools of three different iBONi short interfering RNAs (siRNA) for Cldn3 or 4. For siRNA knockdown, 3 × 10^5^ cells were seeded in 6-well plates and 24 h later transfected with 50 nM siRNA plus 7 μL Lipofectamine RNAiMAX Reagent (Thermo Fisher Scientific, Waltham, MA, USA). Cells were washed with PBS 48 h or 72 h post transfection and RNA and protein was isolated.

### 2.7. MTT Cytotoxicity Assay

MTT assay was used to test cytotoxicity of recombinant CPE (recCPE) or of optCPE transfection and to test cytotoxicity of released CPE from transfected cells. For sensitivity testing of cell lines towards recCPE 6 × 10^3^–4 × 10^5^ cells were seeded into 96-well plates and 24 h later 0, 50, 100, 150 ng mL^−1^ recCPE was added and incubated for 72 h. For determination of toxicity of released CPE in supernatants of transfected cells, 6 × 10^3^ non-transfected cells were seeded into 96-well plates. After 24 h, 100 µL of supernatants from optCPE-transfected cells were added to respective non-transfected cells and incubated for 72 h. For all cytotoxicity assays, 5 mg mL^−1^ MTT (3-(4,5-dimethylthiazyol-2yl)-2,5-diphenyltetrazolium bromide; Sigma) was added 72 h after CPE incubation. Absorbance was measured in triplicates at 560 nm in a microplate reader (Tecan, Groedig, Austria) and values are expressed as percentage of untreated controls.

### 2.8. CPE ELISA

For quantification of CPE in supernatants 24 h or 48 h after transfection, Ridascreen *Clostridium perfringens* enterotoxin ELISA (R-Biopharm, Darmstadt, Germany) was performed. For this, 4 × 10^5^ cells were seeded into 6-well plates and transfected with pCpG-optCPE or pCpG-mscG2 plasmid-DNA. Recombinant CPE was used as standard at serial dilutions of 0.4–25 ng CPE mL^−1^. Measurements were done in duplicates at 450 nm in the microplate reader (Tecan) and values are expressed as percentage of untreated controls.

### 2.9. Immunocytochemistry, Immunohistochemistry and Immunofluorescence

For immunohistochemistry, 2 × 10^5^ cells were seeded into 4-well chamber slides and after 24 h washed with PBS, fixed in 4% paraformaldehyde (PFA, Pierce Thermo Fisher Scientific) in PBS for 15 min, permeabilized with 0.5% Triton-X in PBS for 10 min and blocked with 1% IgG-free albumin (Sigma Aldrich, Taufkirchen, Germany) and 0.05% Tween-20 in PBS for 1 h. Rabbit anti-human Cldn3 or anti-human Cldn4 antibody (Acris) was added as primary antibody, cells were washed with TBST, then incubated with HRP-conjugated goat anti-rabbit IgG antibody (Promega, Madison, WI, USA), washed again and incubated with diamino-benzidine (DAB, DAKO, Hamburg, Germany). Cells were then washed in ddH_2_O, counterstained with hemalum (Roth) and evaluated in a light microscope (Zeiss, Jena, Germany).

For immunofluorescence, 2 × 10^5^ cells were seeded onto cover slips (Steiner GmbH, Siegen Eiserfeld, Germany), washed, fixed in 4% PFA, quenched with 0.1 M glycine for 20 min and blocked with 1% serum-free albumin, 0.05% Tween-20 for 1 h. The respective primary goat anti-claudin-3 rabbit polyclonal IgG (Abcam, Cambridge, UK), goat anti-claudin-4 rabbit polyclonal IgG (Santa Cruz, Dallas, TX, USA) anti-CPE rabbit polyclonal IgG, (BioRad) was added, cells were washed with TBST and incubated with secondary antibody (goat anti-rabbit-Alexa 488, goat anti-rabbit-Alexa 555, goat anti-rabbit-Alexa 647, donkey anti-goat-Alexa 555 and donkey anti-goat-Alexa 647 antibodies, all from Thermo Fisher). Nuclei were stained with DAPI (Sigma-Aldrich), counterstained with Alexa 555-phalloidin (Thermo Fisher Scientific) and evaluated in a confocal fluorescence microscope (Zeiss).

For analysis of Cldn3/4 expression or CPE expression after gene transfer in the PDX tumors, 3–5 μm thick paraffin embedded (FFPE) tumor sections were deparaffinized, fixed with 4% PFA, quenched with 0.1 M glycine for 20 min, incubated with 3% H_2_O_2_ for 10 min, washed with PBS, permeabilized by 0.2% Triton X-100 and blocked with 1% IgG-free albumin and 0.05% Tween-20 for 1 h. Steps for staining are similar to aforementioned procedure.

### 2.10. In Vivo optCPE Gene Transfer

For establishment of subcutaneous tumors, 1 × 10^6^ cells or pieces of approximately 3 × 3 mm in size of patient derived pancreas cancer PDX tissue were inoculated into the left flank of female NMRI: nu/nu mice (*n* = 5 animals per group). Animals were randomized at tumor volume of 0.3 cm^3^ and intratumoral non-viral in vivo jet injection gene transfer (jet injector, EMS Medical Systems SA, Nyon, Switzerland) was performed in anesthetized animals [35]. For this, 50 µg plasmid DNA of respective vector construct was applied once by 5 injections (1 µg DNA µL^−1^ PBS in 10 µL injection volume). Tumor volumes (TV) were measured at indicated time points and calculated using the formula: TV = (width^2^ × length)/2. Animals were sacrificed and tumors were harvested for further analysis. Body weights, clinical signs and behavior were recorded for all mice twice a week. All experiments were performed in accordance with the UKCCCR guidelines and approved by local authorities (State Office of Health and Social Affairs, Berlin, Germany, approval No. Reg0010/19).

### 2.11. Statistical Analysis

For statistical analyses of the in vitro experiments the Student’s *t*-test and one way-ANOVA test were used. For the statistical analyses of in vivo experiments the non-parametric, unpaired *t*-test was used. Error values for the in vitro experiments are S.D. and for in vivo experiments S.E.M.

## 3. Results

### 3.1. Cldn3/4 Expression in Human Pancreas Carcinoma Cell Lines

**Figure 1:**
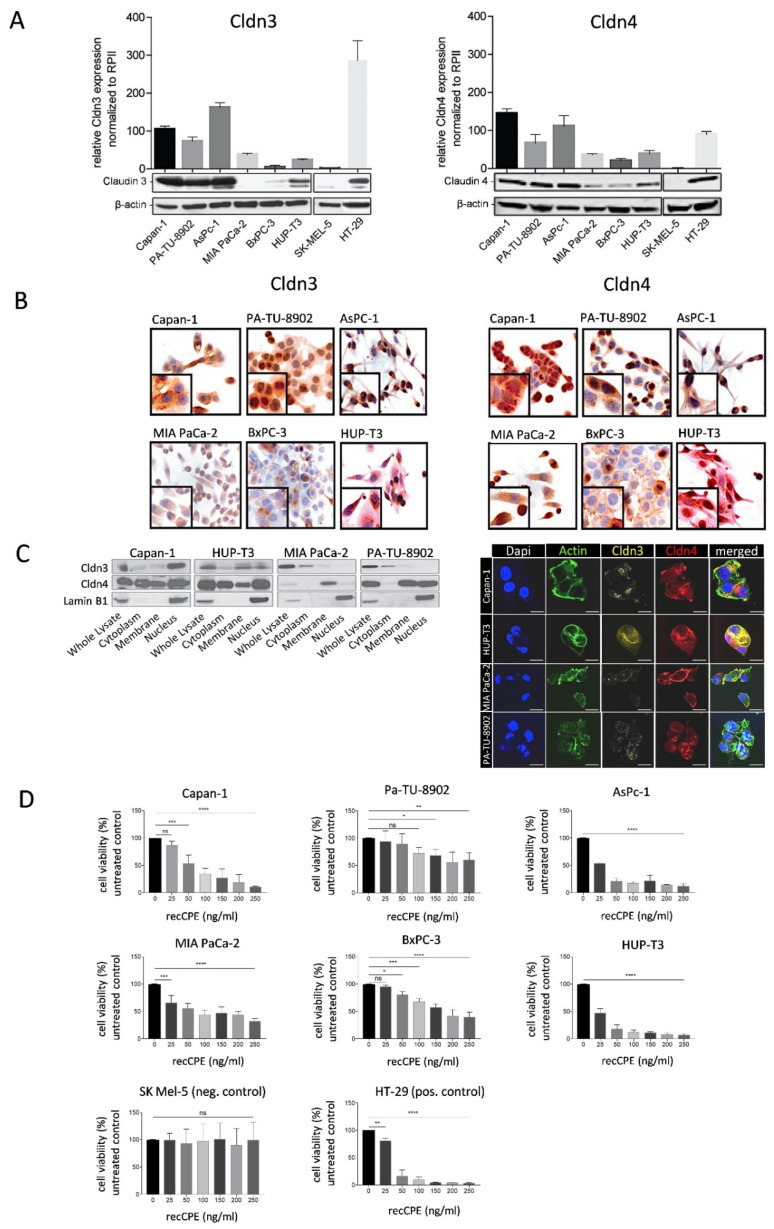
Claudin-3 (Cldn3) and claudin-4 (Cldn4) expression and distribution analysis and sensitivity toward recombinant CPE (recCPE) in human PC cell line panel. (**A**) Quantitative real time PCR (qRT-PCR, graph upper panel) and Western blot analyses (two lower panels for claudin and β-actin, Figure S5) for Cldn3 (left) and Cldn4 (right). The human colorectal cancer cell line HT-29 served as positive control and the human melanoma cell line SK-MEL-5 as negative control with no Cldn3/4 expression. Data are represented as mean values ± S.D. (*n* = 3). (**B**) Representative immunohistochemistry of Cldn3 (left) and Cldn4 (right) in respective pancreatic cancer cell lines, demonstrating different distributions of Cldn3/4 (brown). (**C**) Western blot analysis of cell fractions from four PC cell lines using Cldn3/4, and Lamin B1, showing whole cell lysates, cytoplasmic, membrane and nuclear localization (Lamin B1 marker nuclear fraction, Figure S5). Representative immunofluorescence images (right panel) of respective cell lines, confirming diverse Cldn3 (yellow) and Cldn4 (red) expression in different cell compartments (scale bar = 25 µm). DAPI and actin staining was used for nuclei and cytoplasm, respectively. (**D**) Sensitivity toward recCPE at 72 h post treatment using MTT assay and compared to untreated/solvent treated controls with strong cytotoxic effects in all PC cell lines and positive control line HT-29. SK-MEL-5 negative control cells remained unaffected. MTT data are represented as mean values ± S.D. (*n* = 6), expressed as mean percentage of untreated control. (ns: not significant; * *p* < 0.05; ** *p* < 0.01; *** *p* < 0.001; **** *p* < 0.0001).

Prerequisite for targeted antitumoral oncoleaking activity of CPE is presence of high affinity CPE receptors, such as Cldn3/4, as the CPE-mediated cytotoxicity requires their accessibility. This was analyzed in a panel of human PC cell lines at mRNA and protein level. By this, Capan-1, PA-TU-8902, AsPc-1 and the positive control human colon cancer HT-29 control cells showed high Cldn3/4 mRNA levels, whereas moderate levels of Cldn3/4 were detected in MIA PaCa-2 and HUP-T3 cells (Figure 1A). BxPC-3 cells revealed low Cldn3 expression and moderate Cldn4 expression. The negative control human melanoma cell line SK-MEL-5 did not express Cldn3/4. Western blot, IHC and also immunofluorescence imaging revealed heterogeneous distribution of Cldn3/4 in Capan-1, HUP-T3, MIA PaCa-2 and Pa-TU-8902 cells (Figure 1A–C): apart from membranous presence, both claudins were also detected in the cytoplasm and the nucleus.

### 3.2. Sensitivity of Pancreas Carcinoma Cells toward Recombinant CPE

For sensitivity testing of Cldn3/4 expressing pancreatic cancer cell lines toward recombinant CPE (recCPE) protein, increasing recCPE concentrations from 0 to 250 ng mL^−1^ were applied for 72 h and cytotoxicity was measured (Figure 1D). High Cldn3/4 expressing Capan-1 and AsPC-1 cells as well as HUP-T3 cells with moderate Cldn3/4 expression showed a significant (*p* = 0.0001) dose dependent sensitivity toward recCPE. Despite high Cldn3/4 expression level, PA-TU-8902 cells showed comparatively low sensitivity toward recCPE, since the highest concentration reduced cell viability only to 60% (*p* = 0.0019). By contrast, in BxPC-3 and MIA PaCa-2 cells with low Cldn3/4 expression, higher cytotoxicity was measured. In these cells viability was significantly (*p* = 0.0001) reduced to 40% or 32% compared to control, respectively. However, Cldn3/4 negative cell line SK-MEL-5 was insensitive towards the toxin. RNAi-mediated downregulation of Cldn3/4 expression in MIA PaCa-2 and HUP-T3 cells transfected with siCldn3 and siCldn4 or siCtrl, abolished sensitivity toward recCPE (Figure S2). All this points to the fact, that both claudin expression and accessibility outside tight junctions is important to mediate CPE cytotoxicity.

### 3.3. CPE Gene Transfer Permits Effective and Selective Cell Killing

**Figure 2:**
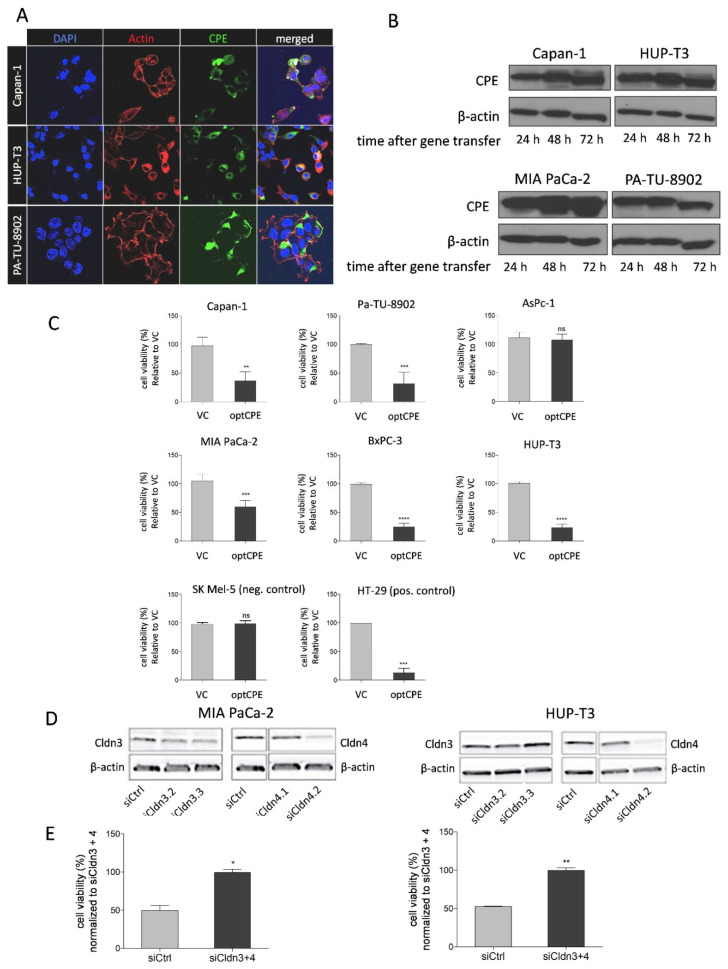
Expression of optCPE and selective cytotoxicity after in vitro gene transfer. (**A**) Representative immunofluorescence images showing expression of optCPE (green) 12 h after transfection in Capan-1, HUP-T3 and PA-TU-8902 cells (40-fold magnification). Cytoplasm was stained with phalloidin (red) and nuclei with DAPI (blue). The images reveal strong CPE expression, with vesicle-like cytoplasmic accumulation, localization on cell membrane and cell contact regions. (**B**) Kinetic of optCPE expression in Capan-1, HUP-T3, MIA PaCa-2 and PA-TU-8902 cells 24 to 72 h after transfection (Figure S6). (**C**) Cytotoxicity of optCPE and vector control (VC) gene transfer was analyzed by MTT assay 72 h after transfection. All transfected Cldn3/4 expressing cells showed significant optCPE toxicity. SK MEL-5 negative control cells remained unaffected, supporting selectivity of optCPE. Data are represented as mean values ± S.D. (*n* = 6), expressed as mean percentage of VC treated cells. (**D**) Rescue experiment in MIA PaCa-2 and HUP-T3 cells transfected with a pool of two specific small interfering RNAs or respective control (siCtrl), leading to efficient knockdown of Cldn3/4 at protein level (Western blot, (Figure S6). (**E**) Cldn-silenced cells were transfected with optCPE. MTT assay was performed 72 h later showing significantly reduced CPE cytotoxicity in MIA PaCa-2 and HUP-T3 cells compared to siCtrl treated cells, demonstrating selectivity optCPE. Data are represented as mean values ± S.D. (*n* = 2), expressed as mean percentage of siCldn3/4 treated cells. (ns: not significant; * *p* < 0.05; ** *p* < 0.01; *** *p* < 0.001; **** *p* < 0.0001).

To evaluate antitumoral efficacy of CPE gene transfer, first presence and expression kinetic for CPE was analyzed. After gene transfer, the immune fluorescence in Capan-1, HUP-T3 and PA-TU-8902 cells showed strong cytoplasmic staining for CPE (Figure 2A). Furthermore, Western blot analyses revealed strong and time-dependent CPE expression starting 24 h after gene transfer and lasting for up to 72 h (Figure 2B). Next, we determined CPE gene transfer mediated selective tumor cell killing in Cldn3/4 positive Capan-1, PA-TU-8902, AsPc-1, MIA PaCa-2, BxPC-3 and HUP-T3 cells as well as the negative control cell line SK -MEL-5 and the positive control line HT-29. The experiments showed significant toxicity by optCPE expression in all Cldn3/4 positive cells with toxicity rates of 40–85%, except for AsPc-1 (Figure 2C). The Cldn3/4 negative cell line SK-MEL-5 remained unaffected after gene transfer, indicating strict claudin-selectivity of optCPE cytotoxicity.

To further prove selectivity of CPE cell killing, RNAi experiments were performed for specific downregulation of Cldn3/4 expression. MIA PaCa-2 and HUP-T3 cells were transfected with a pool of siCldn3 and siCldn4 or siCtrl, respectively (Figure 2D). The downregulation of Cldn3/4 led to reduction of protein expression in both lines 48 h after transfection (Figure 2D), with most pronounced effects for Cldn4 silencing. The Cldn3/4-silenced cells were transfected with optCPE expressing vector and cytotoxic effect was determined 72 h after transfection. By this, optCPE-mediated cytotoxicity was significantly reduced in Cldn3/4-silenced MIA PaCa-2 and HUP-T3 cells compared to siCtrl-transfected cells (*p* < 0.001; Figure 2E). These data support the selective, Cldn-targeted activity of optCPE gene transfer.

### 3.4. Analysis of Rapid Oncoleaking Cell Death Mechanism by CPE Gene Transfer

For the improved and more effective use of the pore-forming toxin, the mode of cell death by CPE gene therapy was of interest. We determined timely activation of apoptotic or necrotic pathways in optCPE-transfected pancreatic cancer cells by live cell imaging for apoptotic signaling, Ca^2+^ influx due to pore formation, activation of caspase-3 and binding of Annexin-V to apoptotic cells. Furthermore, lactate dehydrogenase (LDH) release and calpain activation was determined.

**Figure 3:**
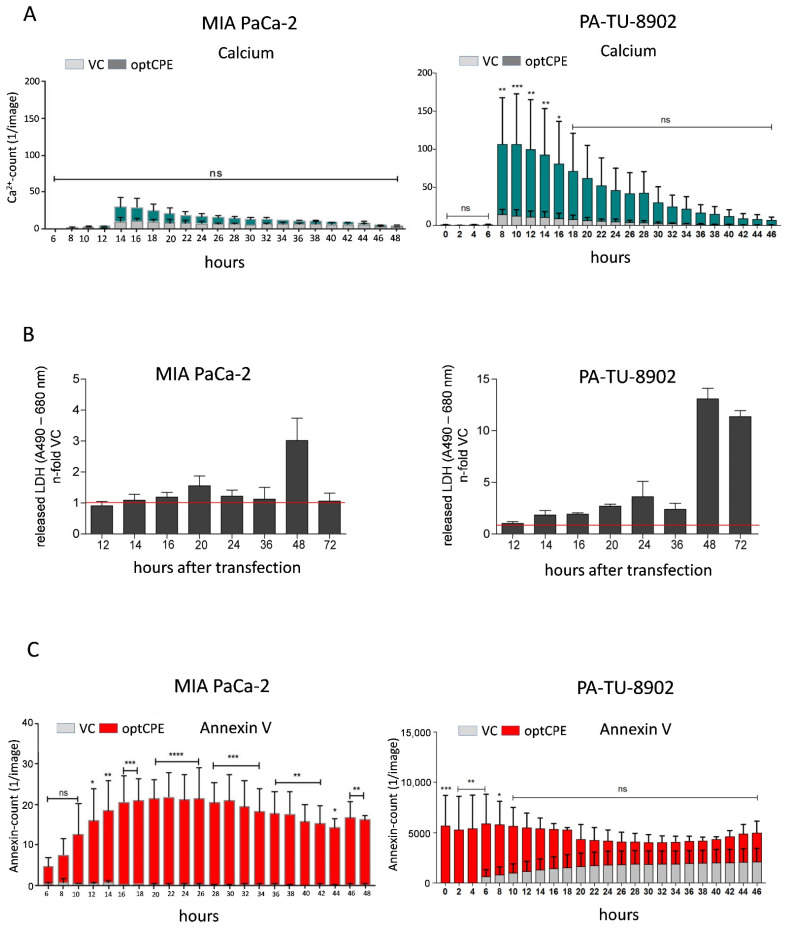

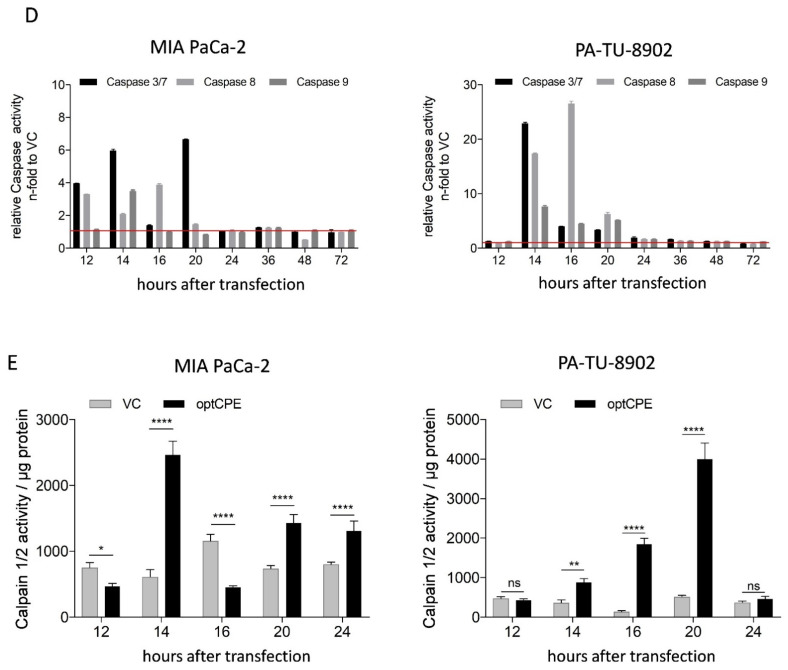
Time course of cytotoxicity in optCPE-transfected human PC cell lines. (**A**) Real-time live-cell analysis by IncuCyte: CPE-mediated cytotoxic effect by analysis of calcium (Ca^2+^) influx (visualized by Fluo-4 AM component) and binding of Annexin-V to apoptotic cells (Annexin V Red Reagent). The optCPE gene transfer led to elevated intracellular Ca^2+^, starting 12 h after transfection, reaching its maximum at 8–14 h, followed by subsequent decreases over time. (**B**) Release of lactate dehydrogenase (LDH): liberated LDH was analyzed in vector control (VC) or optCPE-transfected MIA PaCa-2 (left) and PA-TU-8902 cells (right). The experiment revealed a 1.5-fold and 3-fold increase of LDH release in optCPE expressing MIA PaCa-2 cells 20 h or 48 h after transfection, respectively. PA-TU-8902 cells showed higher LDH release starting 14 h after gene transfer, peaking at 48 h. Data are represented as mean values ± S.D. (*n* = 3), expressed as released LDH, n-fold to VC. (**C**) Real-time live-cell analysis of CPE-mediated cytotoxic effects via binding of Annexin-V to apoptotic cells (IncuCyte Annexin V Red Reagent). (**D**) Activation of caspases in optCPE-transfected PC cells: short-term optCPE action (12–20 h) leads to significant time dependent hyperactivation of all analyzed caspases starting 12–14 h after transfection, with subsequent decrease compared to VC-transfected cells. Data are represented as mean values ± S.D. (*n* = 3), expressed as relative caspase activity n-fold to VC. (**E**) Analysis of calpain-1/2 activation in optCPE expressing PC cell lines. Both lines revealed significant high calpain-1/2 activation 14–24 h after optCPE transfer. Data are represented as mean values ± S.D. (*n* = 3), expressed as calpain-1/2 activity/µg protein. (ns: not significant; * *p* < 0.05; ** *p* < 0.01; *** *p* < 0.001; **** *p* = 0.0001).

The first indication for optCPE action is uncontrolled calcium influx. In MIA PaCa-2 and PA-TU-8902 cells optCPE gene transfer induced rapid calcium influx indicating rapid pore formation between 8 and 14 h after gene transfer, although to different extents in the two lines (40- to 100-fold increase; Figure 3A). Further, in both cell lines LDH is released after optCPE gene transfer with highest values (3- to 13-fold) at 48 h (Figure 3B). These two parameters strongly indicate membrane leakiness and disruption. Such optCPE activity is paralleled by initiation of apoptotic events. In this context, we observed a dramatic increase in annexin V positivity within 6 h after gene transfer in the two lines as a strong indicator for apoptosis (Figure 3C). This was further supported by increased caspase 3/7, −8 and −9 activation as early as 12 to 14 h after the gene transfer (Figure 3D). This increase in caspase activation was also accompanied by the increased calpain 1/2 activity. This results in strong activation of cell apoptosis and necrosis and associates with the dramatic calcium influx (Figure 3E). This was further supported by apoptosis array analysis showing upregulation of not only pro-caspase 3 but also cytochrome C, SMAC/diabolo, Bax, etc. (Figure S3). All these cellular events point to the effective, rapid activation of apoptotic signaling by optCPE gene transfer leading to the efficient killing of pancreatic cancer cells.

### 3.5. Mechanism of Bystander Effect in CPE Gene Therapy

**Figure 4:**
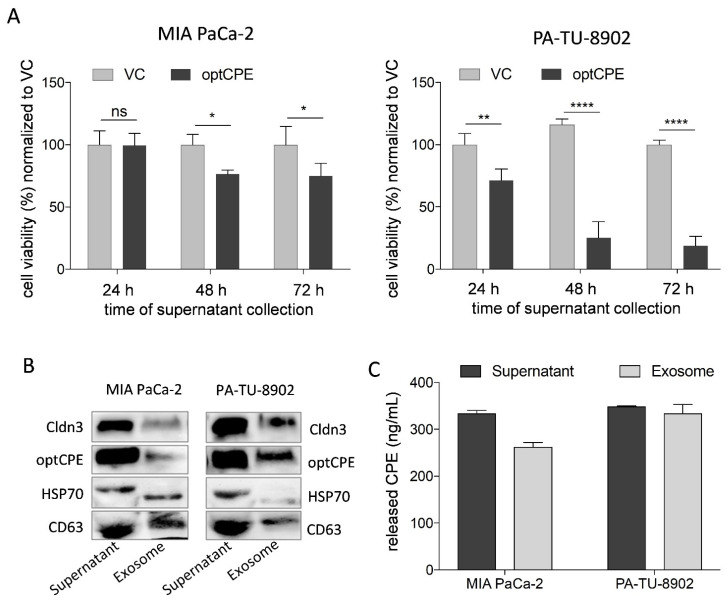
Analysis of bystander effect and of CPE release via exosomes in optCPE-transfected PC cells. (**A**) Analysis of cytotoxicity of released optCPE. Media of vector control (VC) or optCPE-transfected MIA PaCa-2 and PA-TU-8902 cells collected at 24 h, 48 h and 72 h were added to respective non-transfected cells. After 72 h cytotoxicity was determined by MTT. Significant reduction of viability in PA-TU-8902 and MIA PaCa-2 cells was induced by supernatants collected 48 h and 72 h after transfection. For PA-TU-8902 cells, higher toxicity was observed than in MIA PaCa-2 cells. This correlated with the released optCPE amount. (**B**) Western blot of supernatant and exosomes (specific exosomal marker heat shock protein 70, HSP70 and tetraspanin CD63, Figure S7) from transfected MIA PaCa-2 and PA-TU-8902 cells. (**C**) Quantification of optCPE within supernatant and exosomes of optCPE-transfected MIA PaCa-2 and PA-TU-8902 cells by CPE ELISA revealed concentrations of 324.4 and 382.7 ng mL^−1^ CPE in supernatant and 242.6 and 344.5 ng mL^−1^ CPE in exosomes, respectively. Data are represented as mean values ± S.D. (*n* = 3), expressed as mean percentage of VC. (* *p* < 0.05; ** *p* < 0.01; **** *p* < 0.0001).

The transfection experiments revealed much higher optCPE toxicity than anticipated by transfection rates of 10–65% of the human PC cell lines. This suggests that non-transfected cells are potentially affected by a bystander effect of released optCPE. To prove this, supernatants of optCPE-transfected cells were examined. First, we analyzed cytotoxicity of the supernatants released 24 h, 48 h and 72 h after optCPE transfection of PA-TU-8902 and MIA PaCa-2 pancreas cancer cells (Figure 4A). By this, viability of PA-TU-8902 cells dramatically dropped to 25% and 18%, respectively. MIA PaCa-2 cell viability dropped to only 75%. These results correlate with optCPE amounts in the supernatants. High amounts of released optCPE in supernatant of PA-TU-8902 cells (24 h = 441.15 ng mL^−1^, 48 h = 614.25 ng mL^−1^, 72 h = 661.97 ng mL^−1^) induced strong cytotoxicity. The lower toxin amount, released by MIA PaCa-2 cells (24 h = 277.51 ng mL^−1^, 48 h = 320.43 ng mL^−1^, 72 h = 272.24 ng mL^−1^) resulted in lower cytotoxicity.

To determine if optCPE is released via exosomes, media of optCPE-transfected MIA PaCa-2 and PA-TU-8902 cells were used for exosome isolation. Successful isolation was controlled by specific exosomal marker heat shock protein 70 (HSP70) and tetraspanin CD63 in the Western blot (Figure 4B). Both, supernatants and enriched exosomes showed optCPE presence. Quantification of optCPE revealed CPE concentrations of 324.4 and 382.7 ng mL^−1^ in supernatant of transfected MIA PaCa-2 and PA-TU-8902 cells and 242.6 and 344.5 ng mL^−1^ CPE in exosomes, respectively (Figure 4C). This supports the CPE release from transfected cells via exocytosis as well as exosomes, which in turn mediates the bystander effect. All these data clearly support the efficiency of optCPE gene therapy, mediated by prolonged toxin presence and the bystander effect, permitted by CPE release for cancer cell eradication.

### 3.6. In Vivo Kinetic and Antitumoral Effects of CPE In Vivo Gene Transfer

**Figure 5:**
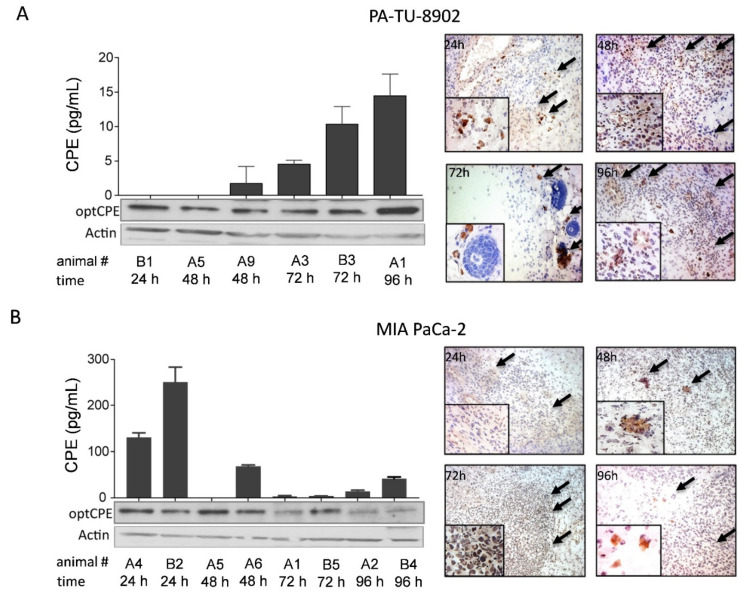

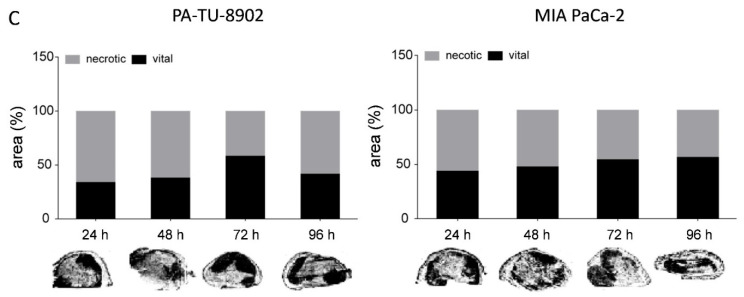
Analysis of optCPE expression in CDX tumor tissue after non-viral in vivo jet-injection gene transfer. (**A**) Time dependent optCPE expression after in vivo gene transfer was analyzed in PA-TU-8902 or MIA PaCa-2 CDX. At indicated time points mice were sacrificed and tumors removed. Western blot analysis (Figure S8) and CPE quantification via CPE ELISA revealed in vivo optCPE expression in both models at all time points (left panel), supported by CPE-specific immunohistochemistry (right panel). (**B**) MIA PaCa-2 tumors demonstrated strong optCPE expression 24–48 h after gene transfer (Figure S8), confirmed by brown staining of CPE-specific immunohistochemistry (right panel, indicated by arrows, 20-fold magnification). ELISA data are represented as mean values ± S.E.M. (**C**) Necrotic areas of optCPE-transfected tumors were analyzed. Tissues were HE stained and ratios of vital vs. necrotic areas were quantified for indicated time points. PA-TU-8902 and MIA PaCa-2 CDX tumors showed massive necrotic areas (grey) surrounded by residual vital tumor tissue (black).

For analysis of time dependent expression and dispersion of optCPE after intratumoral in vivo gene transfer the CDX models PA-TU-8902 or MIA PaCa-2 were used. For this, mice were jet-injected intratumorally with optCPE expression vector and 24, 48, 72 and 96 h after gene transfer animals were sacrificed and optCPE gene expression was determined by Western blot, CPE-ELISA and IHC (Figure 5A,B). Western blot analysis of PA-TU-8902 tumors showed moderate optCPE expression 24 h after gene transfer with peak at 96 h. This was confirmed by quantification of expressed optCPE with values of 0 ng mL^−1^ at 24 h to 290 ng mL^−1^ optCPE after 96 h (Figure 5A,B). MIA PaCa-2 tumors showed high optCPE expression from 24 h after gene transfer on (between 130–250 ng mL^−1^ optCPE), which decreased over time to 2–14 ng mL^−1^ optCPE (Figure 5A,B). IHC further validated the intratumoral presence of expressed optCPE (brown staining). Examination of all tumors via HE staining showed massive tissue destruction within the transfected tumor. This is reflected by the ratio of vital vs. necrotic tissue areas (Figure 5C), indicating more than 50% necrosis of tumors as early as 24 h after gene transfer.

### 3.7. Oncoleaking Efficacy of In Vivo CPE Gene Transfer

**Figure 6:**
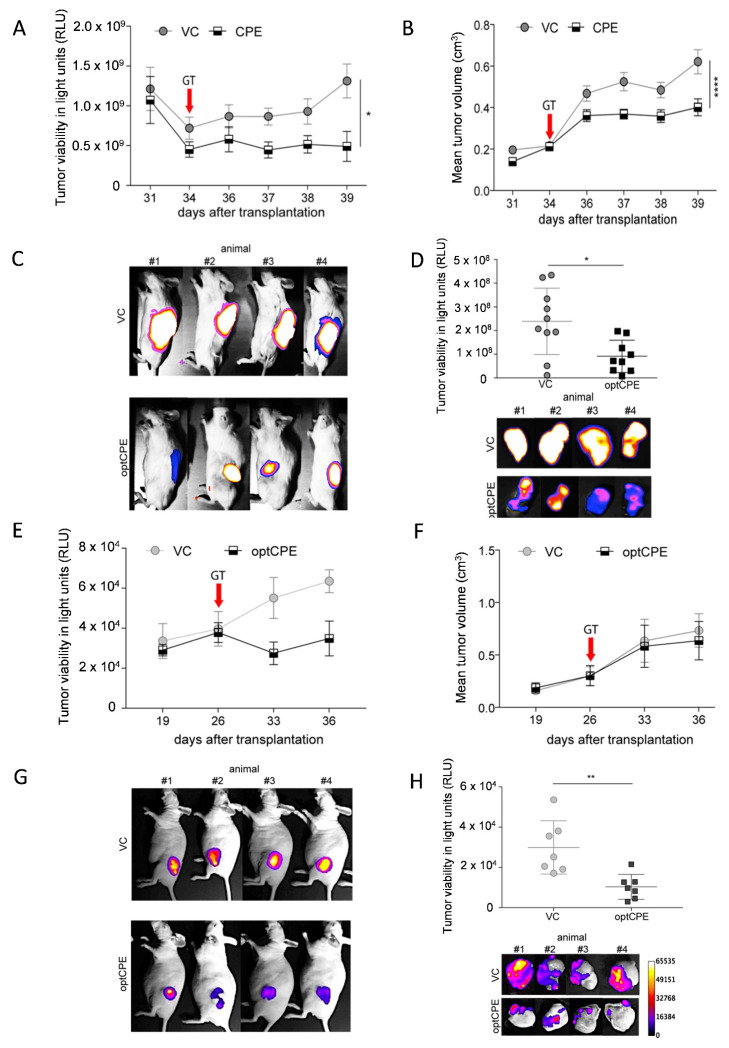

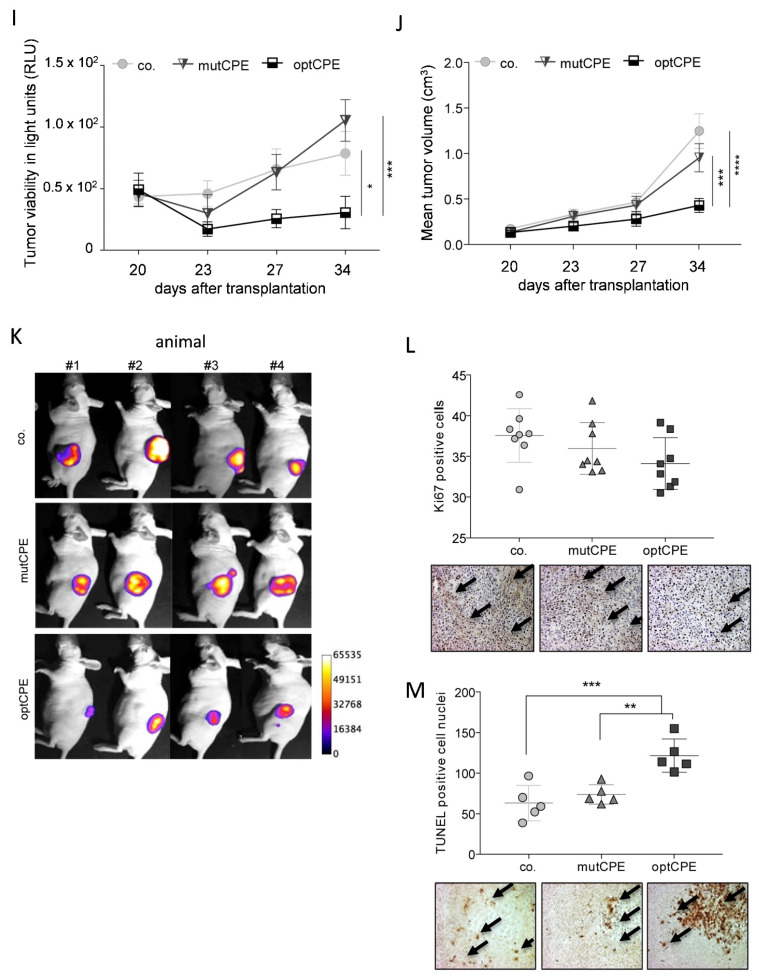
Therapeutic efficacy of non-viral in vivo optCPE gene transfer. (**A**) 34 days after cell injection of optCPE gene transfer (GT) was performed. By this, significant inhibition of tumor viability (determined by bioluminescence; BL), and (**B**) tumor growth (measured via mean tumor volume) was observed in MIA PaCa-2/eGFP-Luc bearing mice compared to VC-transfected animals. (**C**) Decrease of BL in optCPE-transfected tumors compared to controls, shown in representative images. (**D**) Ex vivo tumor BL showed significantly reduced tumor viability at day 39. Data represent mean values ± S.E.M. (*n* = 10). (**E**) Reduction of tumor viability of Capan-1/eGFP-Luc CDX tumors (determined by tumor BLI) was observed after intratumoral optCPE gene transfer (performed 26 days after cell injection). (**F**) Mean tumor volume was not affected by intratumoral optCPE expression. (**G**) Decrease of BL in optCPE-transfected CDX tumors compared to respective vector control (VC), shown by representative images. (**H**) At day 36 ex vivo tumor BL was determined (*n* = 7), showing significantly reduced viability. (**I**) After intratumoral optCPE gene transfer (performed 23 days after tumor cell injection), significant antitumoral efficacy was shown in PA-TU-8902/eGFP-Luc CDX-bearing mice. Tumor viability (determined by tumor BL) decreased significantly in optCPE-expressing tumors compared to empty vector control (VC) or the non-Cldn3/4 binding CPE (mutCPE) expressing tumors. (**J**) Similarly, in vivo optCPE gene transfer significantly inhibited of PA-TU-8902/eGFP-Luc CDX tumor growth compared to VC or mutCPE gene transfer, respectively. (**K**) Tumor BL revealed reduced tumor viability by reduction of BL signals in optCPE-transfected CDX tumors compared to respective controls, shown in representative images. (**L**) Ki67 analysis (brown staining, indicated by arrows) shows decrease in optCPE-transfected groups (not significant). (**M**) In optCPE-transfected PA-TU-8902/eGFP-Luc CDX tumors TUNEL assay shows significantly increased number of TUNEL positive nuclei (brown staining, indicated by arrows) compared to mutCPE or VC-transfected tumors. All data represent mean values ± S.E.M. (*n* = 8). The level of significance was determined using One way ANOVA based on one characteristic or factor (different treatments) or Two way-ANOVA based on multiple characteristics (e.g., different treatments at different time points) and Turkey’s multiple comparison post-test or the nonparametric Mann-Whitney U-test based on comparison of two groups. (n.s. not significant; * *p* < 0.05; ** *p* < 0.01; *** *p* < 0.001; **** *p* = 0.0001).

Next, we investigated the antitumoral effect in high and low Cldn3/4 expressing human pancreatic CDX models. In the Cldn3/4 low expressing MIA PaCa-2/eGFP-Luc tumors non-viral optCPE gene transfer led to significant reduction of tumor viability (*p* = 0.0336) and tumor volume (*p* < 0.0001) compared to vector control (VC) treated mice (Figure 6A,B). This was also reflected by reduced in vivo and ex vivo bioluminescence signals in optCPE expressing tumors (Figure 6C,D). In high Cldn3/4 expressing Capan-1/eGFP-Luc CDX tumor bearing mice jet-injection optCPE gene transfer also led to significant reduction of tumor viability reflected by the reduced tumor bioluminescence signals in vivo and ex vivo (Figure 6E,G,H). By contrast, tumor volume seemed to be unaffected (Figure 6F). Based on our previous observation for CPE action this points to rapid and massive necrosis in these tumors with reduced viability and slightly changed tumor volumes.

In summary, intratumoral optCPE gene transfer exerted significant antitumoral activity. This leads to decreased tumor viability and increased tumor growth inhibition in the human pancreatic cancer CDX models with comparable degree, independent of the Cldn3/4 expression level.

### 3.8. In Vivo Selectivity of CPE Gene Therapy and Mechanism of In Vivo Action

To address selectivity of in vivo optCPE action, an expression plasmid with mutated optCPE (mutCPE, Figure S1; generated by side directed mutagenesis) was used as control. This mutCPE has almost no binding affinity to Cldn3/4 and supports claudin-selectivity of CPE action [32,33]. In PA-TU-8902/eGFP-luc s.c. tumor bearing mice non-viral intratumoral jet-injection gene transfer was performed and tumor viability was measured by bioluminescence. In optCPE-transfected tumors bioluminescence was significantly reduced compared to mutCPE (*p* = 0.0257) or VC (*p* = 0.0003)-transfected tumors (Figure 6I–K). This was supported by significant tumor growth inhibition in optCPE-transfected tumors compared to VC (*p* < 0.00001) and mutCPE (*p* = 0.0004)-transfected tumors. The mutCPE-mediated effects were not different from the VC-transfected group. This validates the strict Cldn3/4 selectivity of optCPE cytotoxicity after intratumoral in vivo gene transfer as important feature for targeted gene therapy.

Since knowledge on the mechanism of action of intratumorally expressed optCPE is limited, the potential induction of apoptosis or necrosis in the treated tumors was analyzed. In this regard, Ki67 reactivity was determined by IHC staining in gene-transfected tumor tissue to evaluate proliferation inhibition (Figure 6L). By this, no significant difference in Ki67 activity was detected between VC, mutCPE or optCPE-transfected tumor tissue. Therefore, optCPE has no impact on cell proliferation in the PA-TU-8902/eGFP-Luc CDX tumors. Further, to detect in situ fragmented DNA as key characteristic of apoptosis, terminal deoxynucleotidyl transferase dUTP nick end labeling (TUNEL) assay was performed. By this, a significantly increased number of TUNEL positive nuclei was determined in optCPE-transfected PA-TU-8902/ eGFP-Luc tumors compared to mutCPE (*p* = 0.0041) or VC (*p* = 0.0009)-transfected tumors. This was particularly seen in areas, with close vicinity to necrotic areas, which could partially be explained by the optCPE-mediated bystander effect (Figure 6M). All this strongly indicates claudin-selectivity in vivo for induction of tumor apoptosis and necrosis.

### 3.9. Oncoleaking CPE Gene Therapy in Pancreas Carcinoma PDX

**Figure 7:**
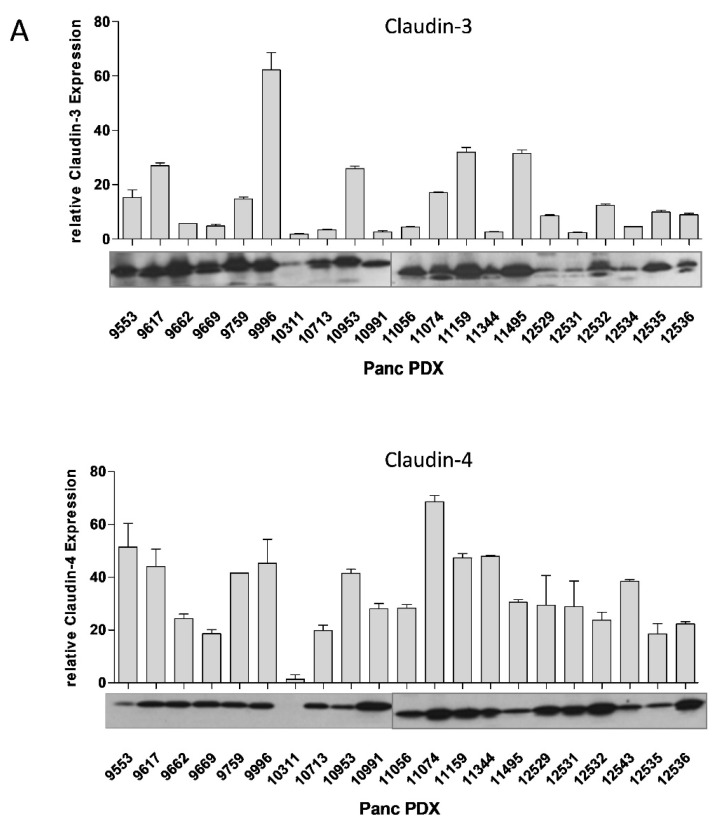

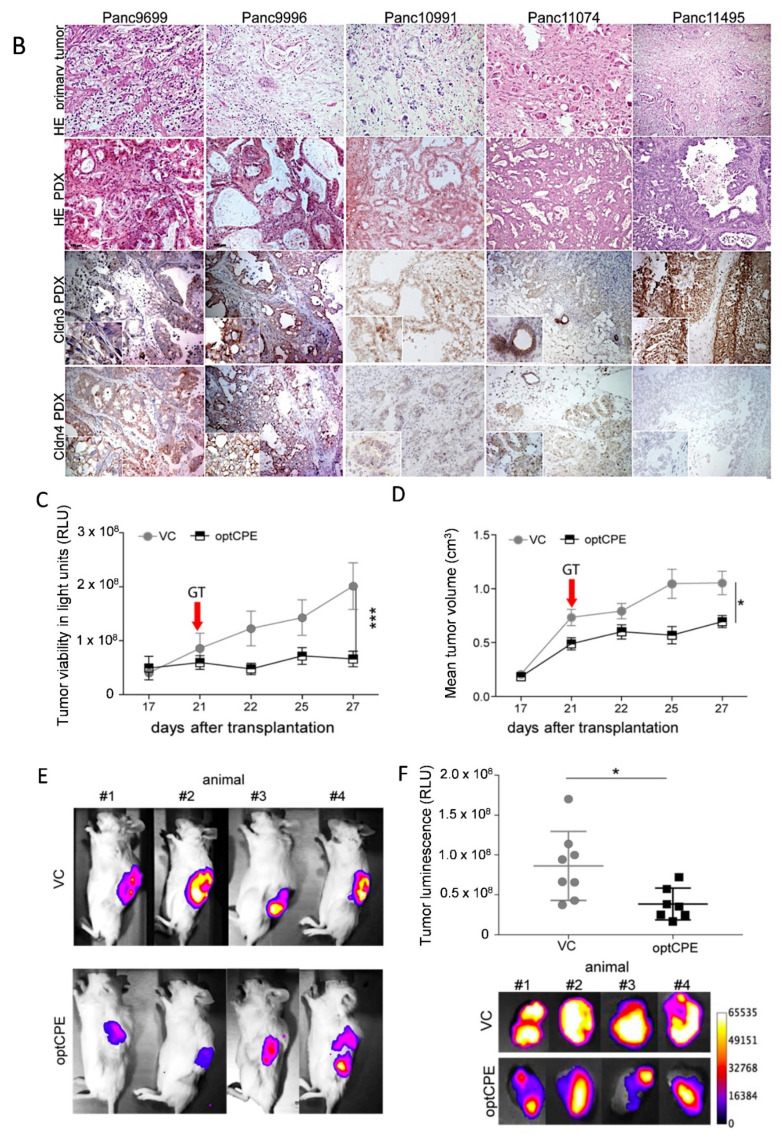

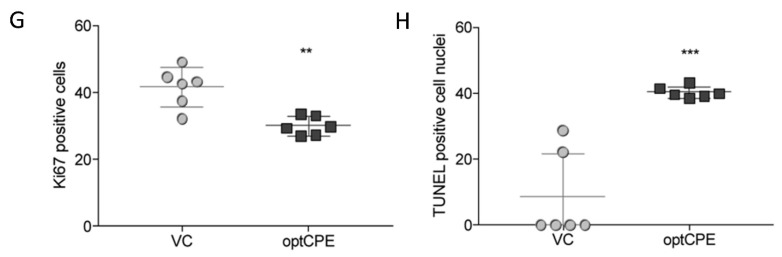
In vivo optCPE gene therapy in patient derived xenograft (PDX) models of PC. (**A**) Claudin3/4 expression analysis in 21 PC patient derived xenograft (PDX) models at mRNA and protein level (Figure S9). (**B**) Histopathology of primary and xenograft tissue show similar histological appearance in representative models (upper two rows). Representative images of Cldn3/4 expression (brown staining) in Panc9699, Panc9996, Panc10991, Panc11074 and Panc11495 PDX tumors (**C**) in optCPE non-viral jet-injection (performed 21 days after PDX derived cell injection) transfected Panc12536/eGFP-Luc-bearing mice significant antitumoral activity was observed. Significantly decreased tumor bioluminescence and (**D**) tumor growth was determined in optCPE-expressing tumors compared to empty vector control (VC). (**E**) In optCPE-transfected tumors reduced luminescence was measured compared to respective vector controls (VC), shown in representative images. (**F**) Significant reduction of viability in optCPE-transfected tumors, determined by quantification of ex vivo bioluminescence of tumors (representative bioluminescence images, lower panel). (**G**) The intratumoral optCPE gene transfer led to significantly reduced Ki67 reactivity within tumor tissue compared to VC treated tissue. (**H**) In optCPE-transfected Panc12536/eGFP-Luc PDX tumors the TUNEL assay revealed significantly increased number of TUNEL positive nuclei compared to VC-transfected tumors. (* *p* < 0.05; ** *p* < 0.01: *** *p* < 0.001).

To extend our gene therapy approach beyond established CDX models, a panel of 21 human pancreatic cancer PDX was characterized regarding Cldn3/4 expression and histopathological features (Figure 7). Primary tumors and respective PDX tissues were analyzed by HE staining. Pancreatic cancer PDX tissues were found to possess similar histological appearance as the corresponding primary tumor tissue: atypical glands, desmoplasia or pleomorphism of nuclei (Figure 7B). PCR expression analysis and Western blot of all 21 PDX models showed Cldn3/4 expression (Figure 7A). This was further confirmed by IHC for Cldn3/4, showing the specific membranous expression within the pancreatic cancer PDX tissue (Figure 7B). This is a strong indication that the target for CPE action is expressed in all of these pancreas cancers.

Next, in vivo experiments were performed using the Panc12536 PDX model, stably transduced with eGFP-Luc luciferase expressing vector. In the in vivo transfected Panc12536/eGFP-Luc bearing mice antitumoral activity of optCPE was observed, since tumor viability (determined by tumor bioluminescence) was significantly reduced in these tumors compared to VC (*p* = 0.0007, Figure 7C–E). This was paralleled by significant tumor growth inhibition (*p* = 0.0142) compared to VC-transfected tumors (Figure 7F). Furthermore, 27 days after gene transfer the ex vivo bioluminescence signal of optCPE expressing tumors showed significantly decreased intensity (*p* = 0.014) compared to VC treated tumors (Figure 7F). This was supported by significantly reduced Ki67 reactivity within the tissue (*p* = 0.0015), particularly for areas in close vicinity to necrosis (Figure 7G). Further, induction of apoptosis was observed in optCPE-transfected tumors, reflected by significantly increased TUNEL positive nuclei (*p* = 0.0002) compared to VC treated control tumors (Figure 7H). Of note, optCPE gene transfer was also shown to be effective in the orthotopic setting, leading to tumor reduction of the Panc12536/eGFP-Luc PDX model (Figure S4).

### 3.10. Combined CPE Oncoleaking Gene Therapy and Drug Treatment

**Figure 8:**
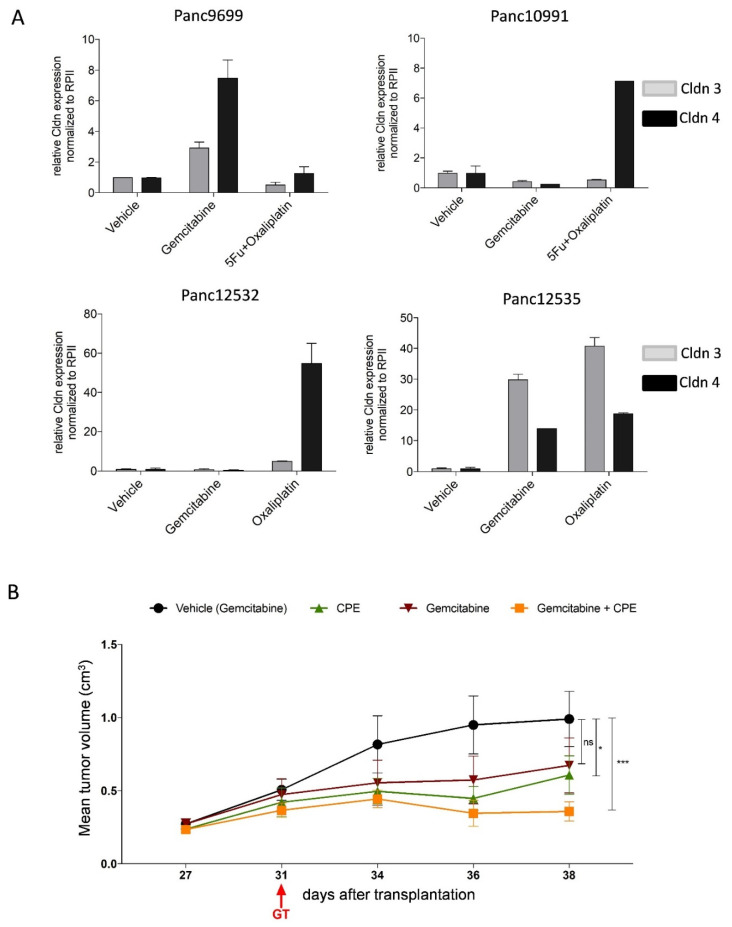
Novel combination of optCPE gene transfer and standard of care drugs. (**A**) Drug treatment induces Cldn3/4 expression in PC PDX, determined in four PDX by qRT-PCR. Gemcitabine treatment increased expression of both Cldns in Panc9699 and Panc12535. Treatment with oxaliplatin or oxaliplatin + 5-FU induces claudin expression in Panc10991 and Panc12532 tumors. Data are represented as means ± S.D. (*n* = 2). (**B**) Synergistic antitumoral effect of combined optCPE gene therapy and gemcitabine treatment in Panc12535 bearing mice. Gemcitabine was applied intraperitoneally at day 27 and four days later optCPE gene transfer (GT) was performed (31 days after tumor transplantation). The in vivo optCPE gene transfer (green) significantly inhibited tumor growth. This was further improved by combination with gemcitabine (yellow) compared to gemcitabine monotherapy (red) or respective vehicle control (black). (ns: not significant; * *p* < 0.05; *** *p* < 0.001).

The oncoleaking gene therapy holds promise for effective combination with conventional therapies of PC. In fact, we addressed such a combinatorial approach in our experiments by using the CPE-based gene therapy in combination with gemcitabine. In this context we analyzed PC PDX treated with the standard of care drugs gemcitabine and oxaliplatin regarding their Cldn3/4 expression. We showed, for the first time, a significant induction of both Cldns in four drug-treated PDX models as first evidence for a drug-induced upregulation of Cldn3/4. Of those, Panc12535 PDX showed the highest claudin upregulation (Figure 8A). Due to this unexpected outcome, we performed an animal experiment with single gemcitabine treatment, optCPE gene transfer or the combination of both to exploit the drug-induced claudin upregulation for therapeutic synergy (Figure 8B). The in vivo study in the Panc12535 model revealed a synergistic effect of combination of optCPE based in vivo gene transfer and gemcitabine treatment. Here, tumor volume was significantly reduced in tumors treated with the combination compared to the respective controls. This indicates that gemcitabine is able to induce Cldn expression, which in turn leads to a better target availability for CPE action. These findings warrant further validation and open new perspectives for clinical use, as gemcitabine resistant tumors could be better eradicated by combination with the novel oncoleaking strategy.

## 4. Discussion

Pancreas carcinoma (PC) is one of the most lethal solid malignancies worldwide [36]. Despite therapeutic advances for PC treatment, the overall survival and prognosis of PC patients at advanced stage remains dismal [37]. Therefore, we developed a novel treatment concept of oncoleaking toxin-based gene therapy. We demonstrated the effective reduction in PC growth in vitro and in vivo by CPE gene transfer, mediated by rapid membrane disruption, associated with tumor cell apoptosis and necrosis.

An increasing number of in vitro and in vivo studies, which use bacterial toxins for cancer treatment (e.g., diphtheria toxin, streptolysin O or *Clostridium perfringens* enterotoxin; CPE) revealed their capability for effective cell killing [10,14]. The establishment of such toxin-based therapy added novel features to cancer treatment, e.g., pore-formation (oncoleaking). Recombinant CPE has demonstrated remarkable and selective cytotoxicity for Cldn3/4 overexpressing epithelial tumors [38,39]. The association of altered Cldn expression and cancer development has been widely reported and there is increasing evidence that dysregulation of tight junction proteins are important features in tumor pathology and carcinogenesis [40]. Elevated Cldn expression level, particularly Cldn3/4, has been reported in various types of solid cancer (e.g., PC, ovarian, breast, prostate and colon cancer) [41,42,43,44,45,46]. Regarding the use of claudins for targeted gene therapy, overexpression of claudin subtypes functioning as high affinity CPE-receptors—such as Cldn3/4—is an important prerequisite. Our analyses for Cldn3/4 demonstrated their overexpression and altered cellular distribution in the panel of human PC cell lines and PDX, with strong membranous and cell–cell contact region, cytoplasmic as well as nuclear presence. This points to an altered cellular distribution, in particular, mainly extrajunctional localization of these claudins, which increases accessibility of these claudins for effective binding of CPE [32]. This is a prerequisite for CPE-based tumor cell killing in vitro and in vivo. In result of this, we determined a dose-dependent cytotoxic effect of recombinant and gene transfected CPE in all PC cell lines, which was positively correlated with the level of Cldn expression. Many studies have described the highly specific interaction of CPE and Cldn3/4, which is essential for selective tumor cell killing [29,30,33,47,48,49]. Selectivity and dependency on claudin availability of CPE-mediated cytotoxicity was supported by our RNAi experiments, where downregulation of the claudins prevents CPE cytotoxicity.

Structure-based mutagenesis enabled design of CPE mutants that also target claudins such as Cldn1 or -5 that are not high affinity receptors for CPE wildtype. Local injection of these recombinant CPE mutants strongly reduced growth of Cldn1-expressing thyroid tumors in CDX models [33]. CPE mutants can be used also for gene transfer to further adjust the targeted claudins to the claudin subtype expression profile of a given tumor entity.

Here we focused on the mechanisms mediated by CPE gene transfer for suicide gene therapy. Our analyses revealed that CPE gene transfer induced rapid fragmentation of nuclei, massive cell swelling, membrane blebbing and formation of membrane vesicles, release of LDH, increased Ca^2+^ influx, activation of caspases and calpain. This suggests, that CPE gene transfer induces different cell death mechanisms, known as “apoptosis-necrosis continuum” [50], which are independent of extent of pore formation. Matsuda et al. and Chakrabarti et al. demonstrated that low CPE doses result in formation of low numbers of membrane pores. This causes limited Ca^2+^ influx [22,51], which triggers caspase-3-mediated cell death by calpain protease activation and results in apoptosis. Two defined factors have been identified that convert an ongoing apoptosis to a necrosis: the availability of intracellular ATP and of caspases [50,52,53]. During apoptosis membrane integrity is maintained until late stages of this process, whereas strong release of LDH is an early event in necrosis [29,30]. Our findings not only support the rapid and effective cell killing by CPE gene transfer via apoptotic and necrotic signaling, they also provide the basis for novel combination approaches to exploit the CPE effects for combination with drugs, which also trigger apoptotic signaling.

For efficient cell killing the bystander effect is supportive in gene therapy. Our immunofluorescence analysis in optCPE-transfected cells revealed vesicle like accumulations as indication for exocytosis. Further, membrane budding was observed, representing a key step in vesicular transport, multivesicular body and exosome formation. Indeed, we showed that CPE is released from cells and is also liberated via exosomes after CPE gene transfer and is acting on neighboring cancer cells. The recent study of Shrestha and colleagues also confirmed a bystander killing mechanism of native and recombinant CPE protein [54]. They demonstrated that supernatants collected from a CPE treated sensitive cell kills other cells that are insensitive toward the toxin. However, they explain this effect by release of factors such as caspase-3 or LDH. By contrast, as we described previously and showed in this study, liberated CPE itself is present in the media of CPE-transfected cells. This is independent of CPE-mediated cytotoxicity or Cldn3/4 expression, as it was also released by the Cldn-negative SK-MEL-5 cells [29,30]. Therefore, two mechanisms contribute to the bystander effect: transmembranous and exosomal release of CPE, which in turn acts on neighboring, non-transfected cancer cells. Such bystander effect is of particular importance for the in vivo use of the CPE gene transfer.

Our study evaluated the anticancer potential of CPE gene therapy in different luciferase-expressing CDX and PDX models. Such locally applied toxin-based gene therapy has been successfully tested for diphtheria toxin in human PSA positive prostate xenograft tumors [55]. In our previous study, we demonstrated selective and efficient antitumoral efficacy of CPE gene therapy in Cldn3/4 overexpressing colon cancer PDX [30]. In this study, CPE gene transfer was effective in inducing massive intratumoral necrosis and induction of apoptosis in tissue areas in close vicinity to necrosis in subcutaneous and also orthotopic CDX and PDX PC models. This is in line with results of others, where necrosis was observed after local in vivo application of recombinant CPE [23,28,33,56,57]. Furthermore, the inclusion of a binding-deficient mutated optCPE (mutCPE) construct (CPE-Y306A/L315A) clearly showed the selectivity of tumor cell killing in vivo pointing to the targeted action of CPE gene therapy. More importantly, in all in vivo CPE therapies we did not observe severe side effects nor body weight losses in the animals. This indicated good tolerability of the CPE gene therapy, as also described previously [28,29,30].

In this study, we also evaluated the potential of CPE gene transfer in combination with drug treatments. In this context, we determined a drug treatment-induced increase of Cldn3/4 expression. Due to this unexpected result, we performed combination studies with gemcitabine and CPE gene transfer, which demonstrated a synergistic effect of this combination. This implied that gemcitabine can induce Cldn expression, which in turn leads to an improved target availability for CPE gene therapy as a basis for new combination approaches in PC therapy.

## 5. Conclusions

In summary we established the efficient use of oncoleaking optCPE suicidal gene therapy in vitro, and more importantly, in subcutaneous and orthotopic human pancreatic cancer CDX and PDX models in vivo. The study emphasizes the great potential of this novel strategy, as it is selective for therapy of pancreas carcinoma.

## Data Availability

The data presented in this study are available on request from the corresponding author.

## Supplementary Materials

The following are available online at https://www.mdpi.com/article/10.3390/cancers13174393/s1, Figure S1: Model of CPE bound to claudin-4, Figure S2: Cldn-dependence of recombinant CPE (recCPE) action in pancreas carcinoma cells, Figure S3: Analysis of impact of optCPE gene transfer on apoptotic signaling by human apoptosis array, Figure S4: Oncoleaking in vivo effect of optCPE suicidal gene therapy in orthotopic human pancreatic cancer PDX model, Figure S5: Original Western blots of Figure 1, Figure S6: Original Western blots of Figure 2, Figure S7: Original Western blots of Figure 4, Figure S8: Original Western blots of Figure 5, Figure S9. Original Western blots of Figure 7, Table S1: Quantitative analysis of CPE release by PC cell lines into cell culture medium at indicated time points after optCPE gene transfer.
