# Supplementary material for: Effective Oncoleaking Treatment of Pancreatic Cancer by Claudin-Targeted Suicide Gene Therapy with Clostridium perfringens Enterotoxin (CPE)
Source: Cancers (Basel). 2021 Aug 31;13(17):4393. doi: 10.3390/cancers13174393 (PMC8431234; doi:10.3390/cancers13174393)
Supplement: Supplementary file 1 [file cancers-13-04393-s001.zip › cancers-1287919-supplementary.pdf]

cancers

MDPI

Supplementary Materials: Effective Oncoleaking Treatment of Pancreatic Cancer by Claudin-Targeted Suicide Gene Therapy with Clostridium Perfringens Enterotoxin (CPE)

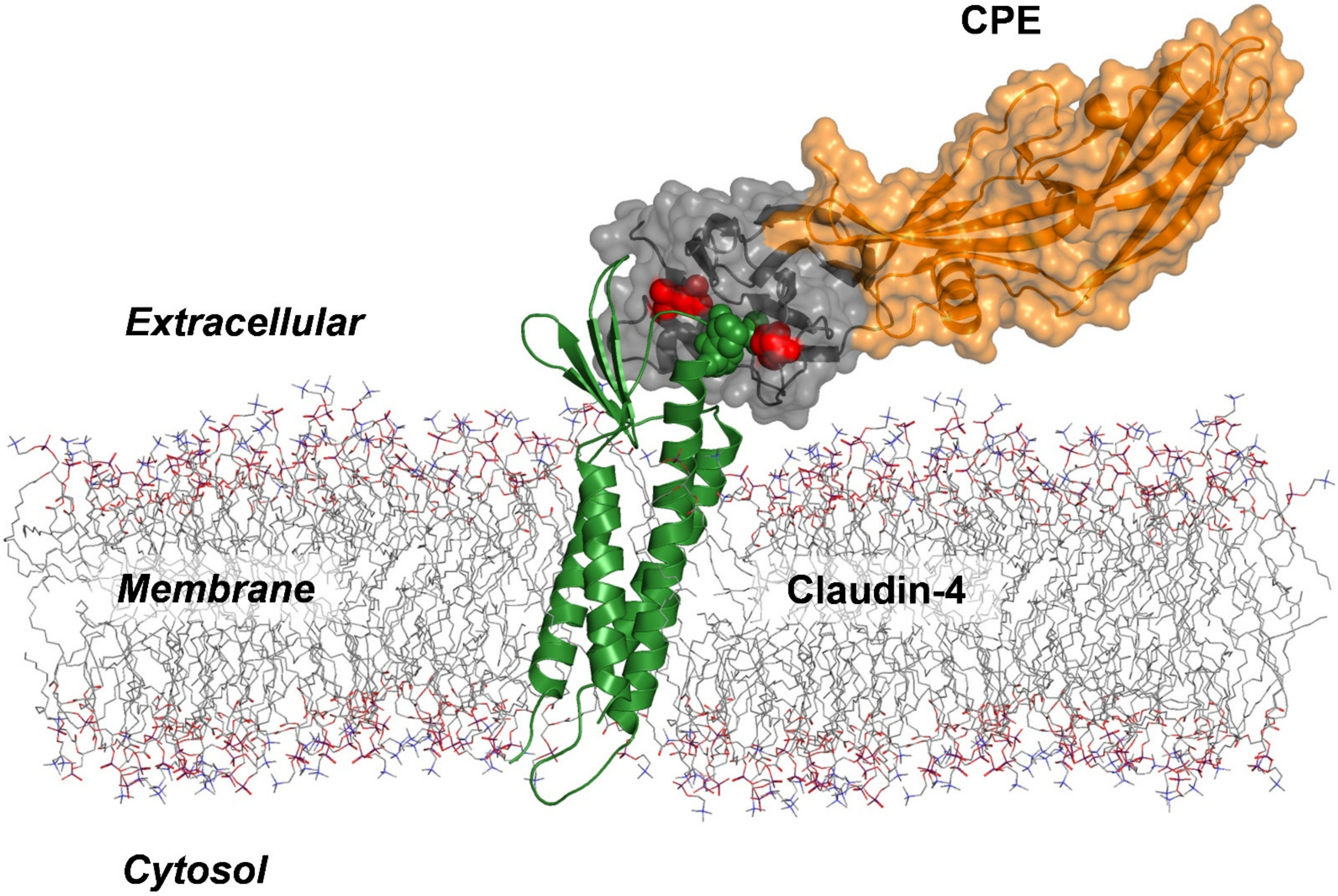

Figure S1. Model of CPE bound to claudin-4. The C-terminal domain of CPE (cCPE, grey) binds to the extracellular domain of claudin-4 (green). Tyr306 and Leu315 residues (red spheres) of CPE are critical parts of the claudin3/4 binding pocket. Claudin-4 residues critical for binding next to Tyr306 are shown as green spheres. Tyr306Ala/Leu315Ala mutation in CPEmut abolishes binding. Claudin-4 is shown as cartoon, CPE as cartoon with transparent surface. Membrane lipids (POPC) are shown as lines. Scheme is based on previous studies [20,21,32,33,34] and the aligned crystal structures of cCPE: claudin-4 complex (PBD ID: 5b2g, [34]) and CPE (PBD ID: 3am2, 20) generated with Maestro and Pymol (Schrödinger).

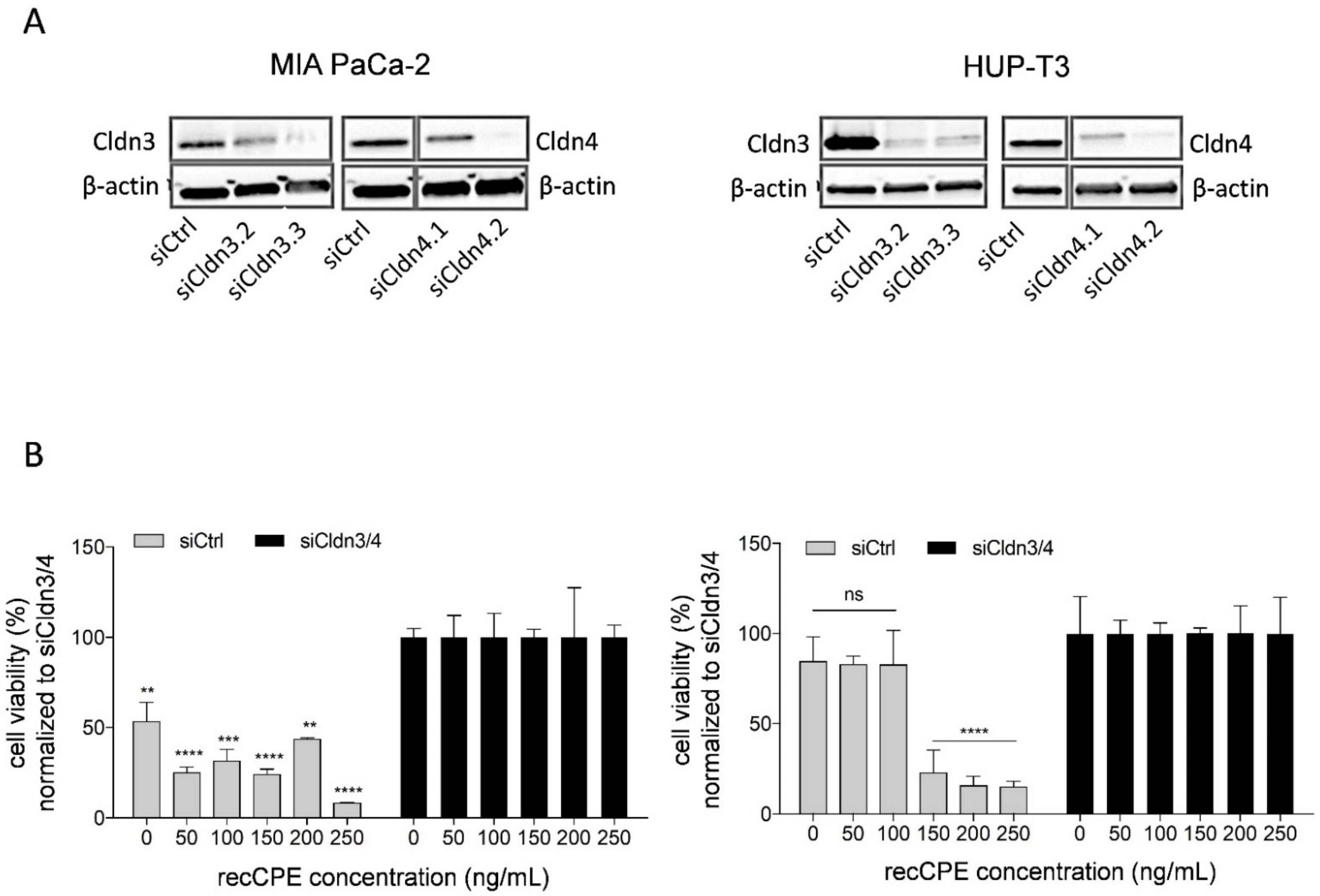

Figure S2. Cldn-dependence of recombinant CPE (recCPE) action in pancreas carcinoma cells. (**A**) Rescue experiment was performed to demonstrate dependency of recombinant CPE activity on Cldn3/4 availability. Representative pancreatic cancer cell lines MIA PaCa-2 and PA-TU-8902 were transfected with a pool of specific small interfering RNA (siRNA) or respective control (siCtrl), leading to efficient knockdown of Cldn3/4 at protein level, demonstrated in representative Western blot. (**B**) Cldn3/4 silenced cells were treated with recCPE at indicated concentrations and MTT assay was performed after 72 h, showing significantly reduced sensitivity of MIA PaCa-2 (left) and PA-TU-8902 cells toward CPE (right). Data are represented as means ±S.D. ($n = 2$) and expressed as mean percentage of siCldn3/4 treated cells. Level of significance was calculated by One way-Anova and Turkey's multiple comparison post-test, ns: not significant; * $p < 0.05$; ** $p < 0.01$; *** $p < 0.001$; **** $p = 0.0001$.

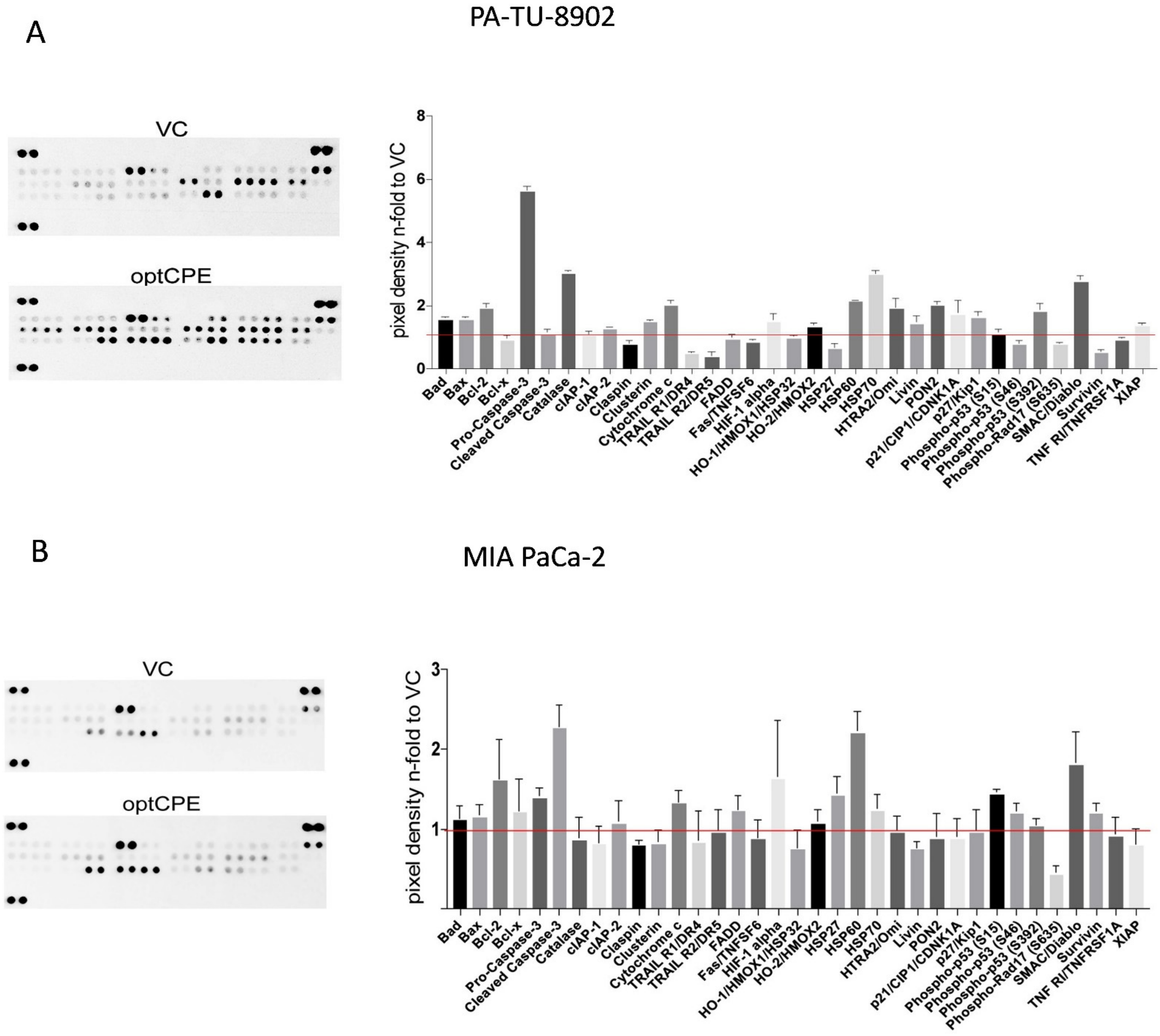

Figure S3. Analysis of impact of optCPE gene transfer on apoptotic signaling by human apoptosis array. (**A**) Transfected PA-TU-8902 cells were harvested 18 h after transfection. Representative array for vector control (VC) or optCPE transfected PA-TU-8902 shows difference in apoptosis related proteins (left panel). Summary of all analyzed proteins, detected on apoptosis array (right panel). The pro-apoptotic proteins Bax, pro-caspase-3, cytochrome c or the stress-induced apoptotic proteins second mitochondria-derived activator of caspases (SMAC/Diabolo) and high temperature requirement protein serine peptidase 2 (HTRA2/OMI) were highly activated compared to vector control (VC) transfected cells. (**B**) Transfected MIA PaCa-2 cells were harvested 26 h after transfection. Representative array for VC or optCPE transfected MIA PaCa-2 cells, demonstrating small difference in apoptosis related proteins (left panel). Summary of all analyzed proteins, detected on apoptosis array (right panel). The pro-apoptotic proteins, cleaved caspase-3, cytochrome c or SMAC/Diabolo were highly activated compared to vector control (VC) transfected cells. All data are represented as means ±S.D. ($n = 2$) and expressed as pixel density. Level of significance was calculated by Student's t-test, ns: not significant; * $p < 0.05$; ** $p < 0.01$; ***p < 0.001; ****p = 0.0001.

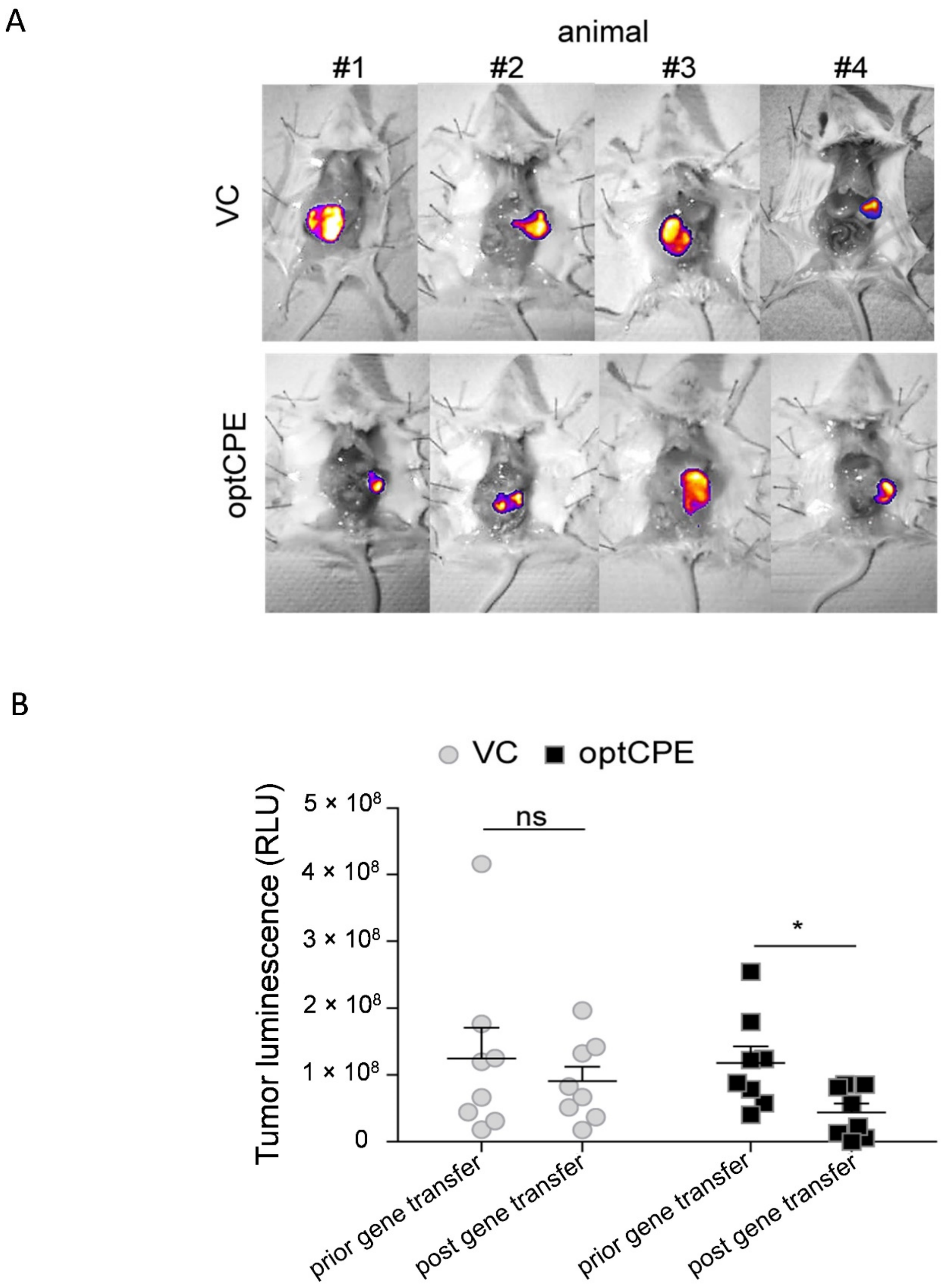

Figure S4. Oncoleaking in vivo effect of optCPE suicidal gene therapy in orthotopic human pancreatic cancer PDX model. Panc12536/eGFP-Luc tumors were generated to analyze antitumoral effect of optCPE at orthotopic tumor site. The non-viral gene transfer was achieved by intratumoral needle injection. (**A**) Bioluminescence of orthotopic PDX in VC treated or optCPE treated mice. **(B)** optCPE generated antitumoral effect, reflected by significantly reduced luminescence signals, while VC transfected tumors remained unaffected. Data represent mean ±S.E.M. (n = 8). Level of significance was determined using the nonparametric Mann-Whitney test, ns: not significant; n.s., significant; * $p < 0.05$.

Figure 1A Western blots: Cldn3 expression in cell lines

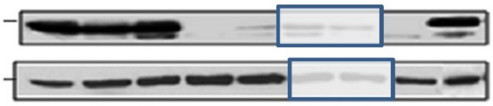

Figure 1A Western blots: Cldn4 expression in cell lines

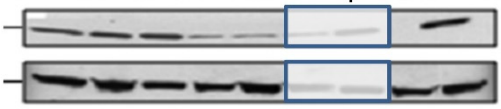

Figure 1C Western blots: Cldn4 & lamin expression In HUP-T3 and Capan-1 cells

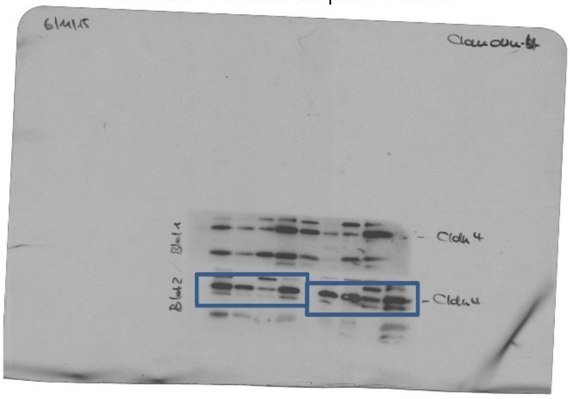

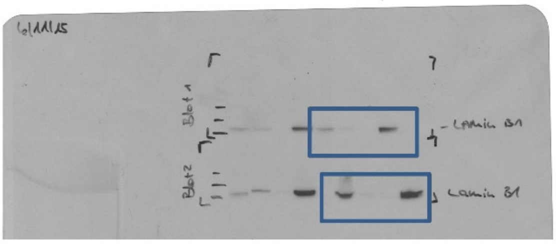

Figure 1C Western blots: Cldn4 & lamin expression In MIA PaCa-2 and Pa-Tu-8902 cells

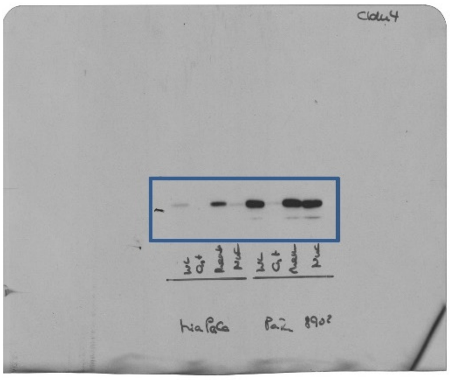

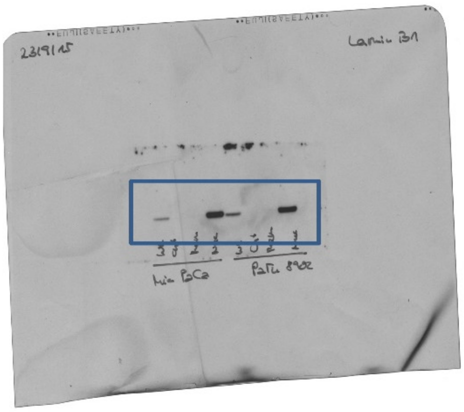

Figure 1C Western blot Cldn3 expression for Capan-1

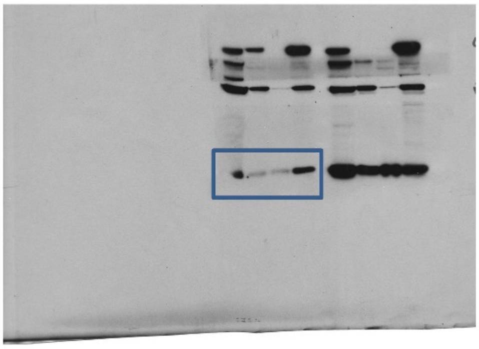

Figure 1C Western blots: Cldn3 expression for MIA PaCa-2

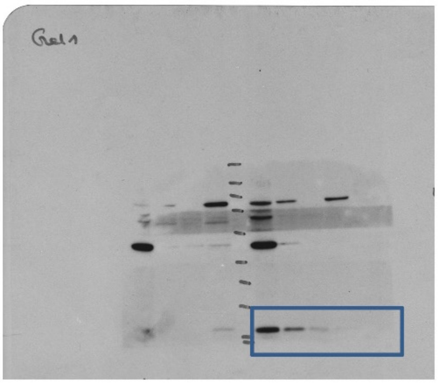

Figure 1C Western blots: Cldn3 expression for PA-TU-8902

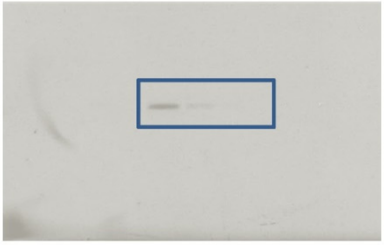

Figure 1C Western blot: Cldn3 expression for HUP-T3

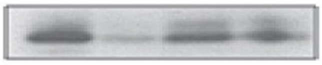

Figure S5. Original Western blots of Figure 1: parts of the blot used in respective figure are marked by frame.

Figure 2 B Western blots: CPE expression for Capan-1, HUP-T3, MIA PaCa-2 and PA-TU-8902

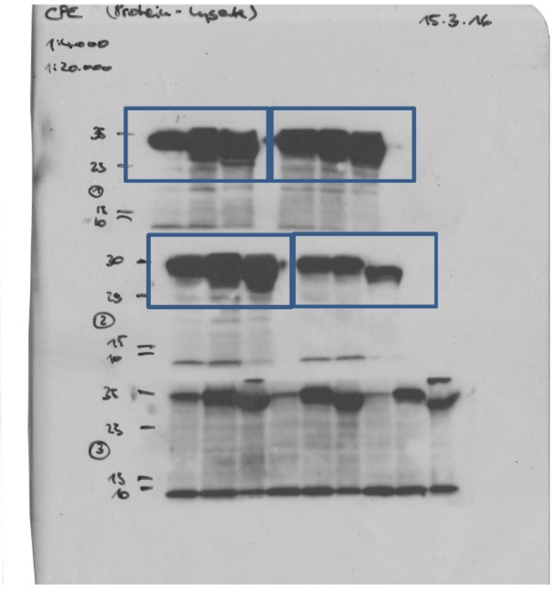

Figure 2 B Western blots: beta-actin expression for Capan-1, HUP-T3, MIA PaCa-2 and PA-TU-8902

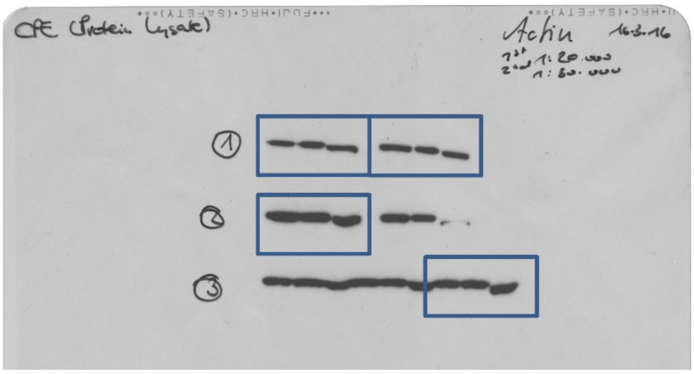

Figure 2D Western blots: MIA PaCa-2 ß-actin, siCtrl and siCldn3.2/siCldn3.3

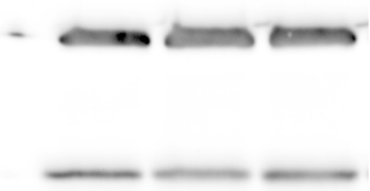

Figure 2D Western blots: MIA PaCa-2 ß-actin and siCldn4.1/siCldn4.2

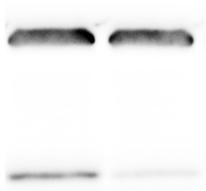

Figure 2D Western blots: HUP-T3 ß-actin, siCtrl and siCldn3.2/siCldn3.3

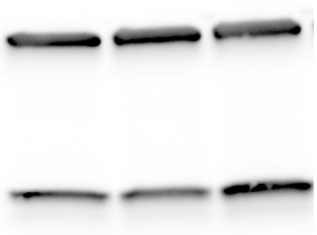

Figure 2D Western blots: HUP-T3 ß-actin and siCldn4.1/siCldn4.2

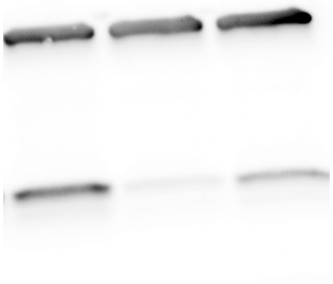

Figure S6. Original Western blots of Figure 2: parts of the blot used in respective figure are marked by frame.

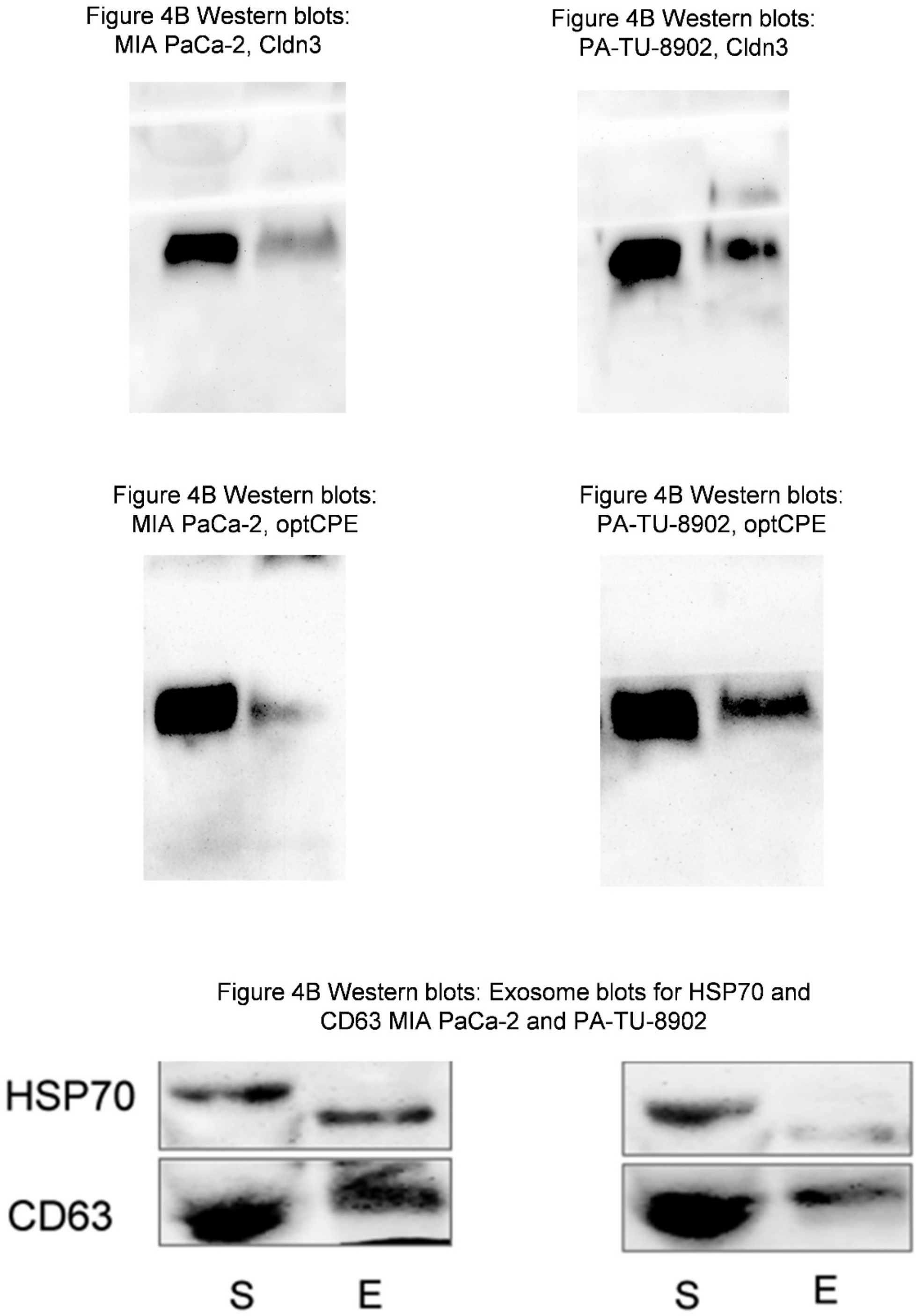

Figure S7. Original Western blots of Figure 4: parts of the blot used in respective figure are marked by frame.

Figure 5A Western blot: CPE in PA-TU-8902 tumors in vivo

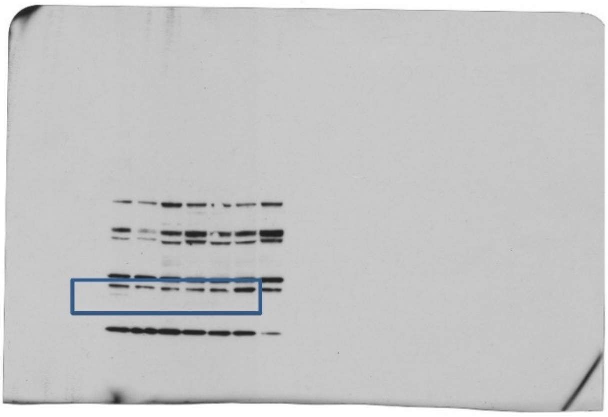

Figure 5A Western blot: beta-actin in PA-TU-8902 tumors in vivo

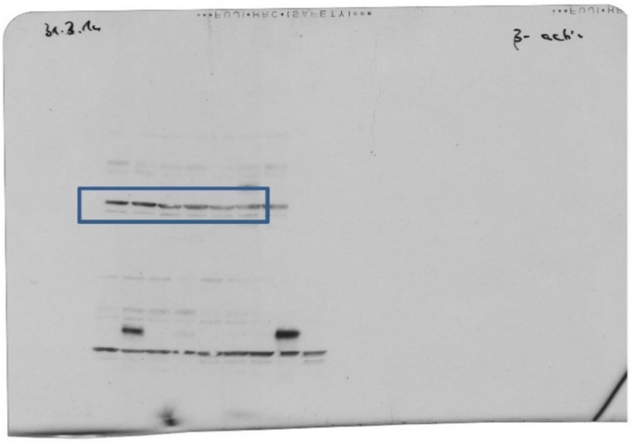

Figure 5B Western blot: CPE in MIA PaCa-2 tumors in vivo

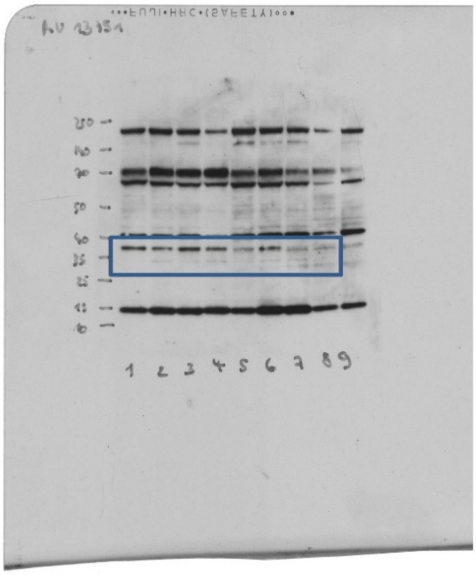

Figure 5B Western blot: beta-actin in MIA PaCa-2 tumors in vivo

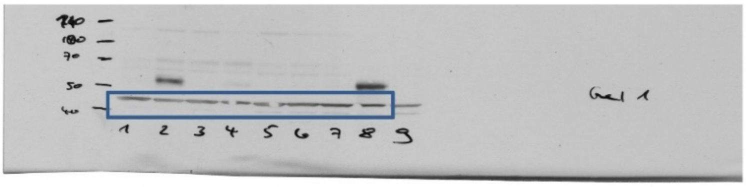

Figure S8. Original Western blots of Figure 5: parts of the blot used in respective figure are marked by frame.

Figure 7A Western blots: Cldn3 expression in pancreas PDX, model 9553 to 10991

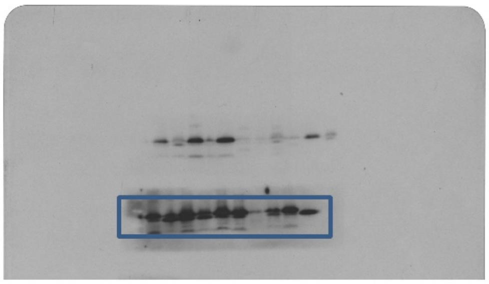

Figure 7A Western blots: Cldn3 expression in pancreas PDX, model 11056 to 12536

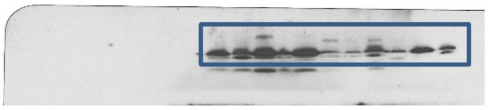

Figure 7A Western blots: Cldn4 expression in Pancreas PDX, model 9553 to 10991 and 11056 to 12536

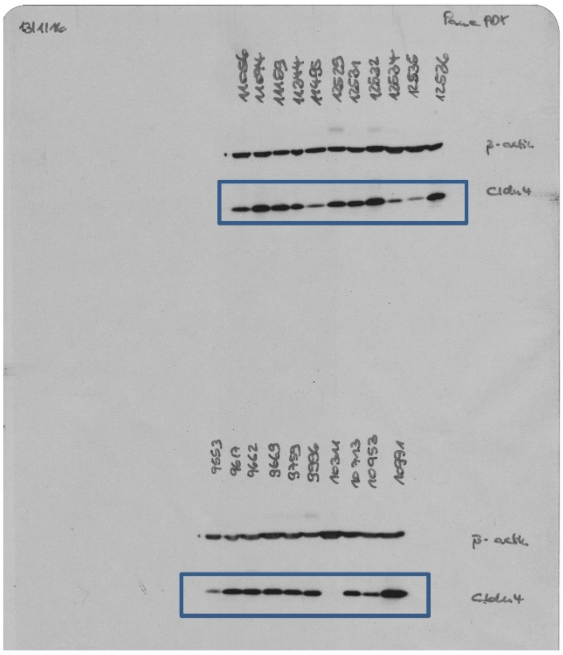

Figure S9. Original Western blots of Figure 7: parts of the blot used in respective figure are marked by frame.

Table S1. Quantitative analysis of CPE release by PC cell lines into cell culture medium at indicated time points after optCPE gene transfer. Supernatants of optCPE transfected PC cells were harvested at indicated time points after transfection and analyzed by specific CPE ELISA for quantification.

Supernatant	12 h	24 h	36 h<br>ng CPE/mL	48 h	72 h
Capan-1	542.45 ± 156.56	612.08 ± 20.15	292.11 ± 137.71	693.29 ± 52.43	677.06 ± 38.66
HUP-T3	19.46 ± 0.69	554.83 ± 24.51	691.72 ± 17.79	128.78 ± 23.32	365.72 ± 11.28
MIA PaCa-2	38.29 ± 10.12	277.51 ± 16.57	616.23 ± 14.38	320.43 ± 22.15	272.24 ± 90.37
PA-TU-8902	663.98 ± 103.75	441.15 ± 30.81	599.07 ± 56.53	614.25 ± 28.24	661.97 ± 23.35

Supplementary Methods:

1. Western Blot Analysis

For protein analysis, cells or tissue cryosections were lysed in RIPA buffer (50 mM TRIS, 150 mM, NaCl, 1% Nonidet P-40, 0.5% sodiumdeoxycholate, protease inhibitor,

ddH_2O) and 25 µg of protein was electrophorezed in 10% precast NuPAGE gels (Invitrogen), 1 h at 180 V and transferred to nitrocellulose membranes (Hybond-C Extra, Amersham, Freiburg, Germany) by semidry blotting (Turbo Blot BioRad, Munich, Germany) at 20 V, 25 min. Membranes were blocked 1 h at room temperature (RT) in TBS (50 mM Tris, 150 mM NaCl, pH 7.5, 5% fat-free dry milk and 2.5% casein) and washed in TBST (0.05% Tween 20 in PBS), 2 × 5 min at RT. As primary antibody rabbit anti-claudin-3 antibody (1:3000, Acris, Germany), rabbit anti-claudin-4 antibody (1:3000, Acris, Germany), rabbit polyclonal anti-CPE (1:4000, BioRad, CA, USA), mouse monoclonal anti-β-tubulin (1:2000, BD Bioscience, USA) or mouse monoclonal anti-β-actin antibody (1:25000, Pierce, MI, USA) was added respectively over night at 4 °C and washed in TBST. As secondary HRP-labeled goat anti-rabbit-IgG antibody (1:10000, Promega, Madison, WI, USA), HRP-labeled goat anti-mouse-IgG antibody (1:25000, Pierce) or goat anti-mouse IgM/IgG (1:10000, Sigma-Aldrich) was added for 1 h, RT. Membranes were washed in TBST. Detection was done using ECL solution (Amersham) and exposure to Kodak X-Omat AR film (Kodak, Stuttgart, Germany).

2. Immununocytochemistry, Immunofluorescence and Immunohistochemistry

For immunohistochemistry 2 × 10^5 cells were seeded into 4-well chamber slide and after 24 h washed with PBS, fixed 15 min in 4% paraformaldehyde (PFA, Pierce Thermo Fisher Scientific) in PBS, permeabilized 10 min with 0.5% Triton-X in PBS and blocked 1 h with 1% IgG-free albumin (Sigma Aldrich, Taufkirchen, Germany) and 0.05% Tween 20 in PBS at room temperature (RT). As primary antibody, rabbit anti-human Cldn3 or rabbit anti-human Cldn4 antibody (1:100, Acris) was added for 2 h at RT. Cells were washed with TBST and incubated with HRP-conjugated goat anti-rabbit IgG antibody (1:500, Promega) for 1 h at RT. Then, cells were washed in PBS, incubated 1 to 5 min with diamino-benzidine (DAB, DAKO, Hamburg, Germany) at RT and washed in ddH_2O for 5 min. Cells were counterstained for 30–60 s with hemalum (Roth), rinsed in tap water, covered with glycergel (DAKO) and evaluated in a light microscope (Zeiss, Jena, Germany).

For immunofluorescence, 2 × 10^5 cells were seeded onto cover slips (Steiner GmbH, Siegen Eiserfeld, Germany). Cells were washed with PBS, fixed 15 min in 4% PFA in PBS, quenched 20 min with 0.1 M glycine in PBS and blocked 1 h with 1 % serum-free albumin and 0.05% Tween 20 in PBS at RT. The respective primary antibody (goat anti-claudin-3 rabbit polyclonal IgG; Abcam, Cambridge, UK; goat anti-claudin-4 rabbit polyclonal IgG; Santa Cruz, TX, USA; anti-CPE rabbit polyclonal IgG, BioRad) was added for 2 h at RT. Cells were washed with TBST and incubated with secondary antibody (goat anti-rabbit-Alexa 488, goat anti-rabbit-Alexa 555, goat anti-rabbit-Alexa 647, donkey anti-goat-Alexa 555 and donkey anti-goat-Alexa 647 antibodies, all from Thermo Fisher) for 1 h at RT. Nuclei were stained with DAPI (Sigma-Aldrich) and counterstaining of cytoplasm was done by using Alexa 555-phalloidin (Thermo Fisher Scientific). Cells were evaluated in a confocal fluorescence microscope (Zeiss).

To detect Cldn3/4 expression or expressed CPE after gene transfer in the PDX tumor samples via immunohistochemistry, 3–5 µm paraffin embedded tumor sections were deparaffinized, fixed with 4% PFA for 15 min at RT, quenched 20 min with 0.1 M glycine, incubated 10 min with 3% H_2O_2, washed with PBS, permeabilized by 0.2% Triton X-100 in PBS for 10 min, RT and blocked 1 h with 1% IgG-free albumin and 0.05% Tween 20 in PBS at RT. Following steps of staining are according to aforementioned procedure.

3. Transduction to Generate Stably eGFP-Luc Expressing Cancer Cell Lines

1.5 × 10^7 HEK cells were plated for transfection. After 24 h cells were transfected using 2.85 mL serum-free medium, mixed with 90 µg of polyethylenimine (PEI) and kept at room temperature for 5 min. 30 µg of eGFP-Luc lentiviral plasmids with packing vectors (20 µg psPax2, 10 µ pMD2. G) were mixed and incubated at room temperature for 20 min and added to the respective plates. After 48 h of incubation, the supernatant was collected

and filtered (0.45 µm filter). The filtered supernatant was loaded on a 20 % sucrose cushion and centrifuged at 4 °C for 4 h at 28,000 rpm. The viral particles were dissolved in 500 µL sterile PBS and stored at −80 °C until use. Capan-1, HUP-T3, MIA PaCa-2 and PA-TU-8902 cells were transduced in 6-well plates with a multiplicity of infection (MOI) less than 10 for each respective well. After 24 h of incubation, virus-containing medium was replaced with the regular medium and the GFP expressing cells were sorted using FACS. After sorting, the luciferase expression of the stably transduced cells was analyzed by measuring relative luminescence using the Steady-Glo® Luciferase Assay System (Promega). The eGFP-Luc stably expressing cells were used for animal experiments and bioluminescence imaging, respectively.

4. Generation of Patient Derived Xenograft (PDX) Models of Pancreatic Cancer

For in vivo expression and therapy studies, 21 pancreatic cancer PDX models, established and kindly provided by EPO Berlin Buch GmbH. Tumor staging, grading and typing was performed according to UICC and WHO guidelines. Tumor tissue of patients was collected after surgery, shortly stored in RPMI medium supplemented with 10 % FBS and antibiotics, diced into 15–20 mm^3 pieces and implanted into subcutaneous pocket on one side of the lower back into anesthetized immunodeficient mice. After successful engraftment, tumors were passaged and expanded in large cohorts for in vivo experiments.

5. Apoptosis Assay by Annexin-V/Propidium Iodide (PI) Staining and FACS Analyses

To elucidate CPE-mediated mechanisms of cell death, Annexin-V / PI stainig (Annexin V-FITC Apoptosis Detection Kit, Abcam) was performed and analyzed by FACS. Staining with Annexin-V and PI allows identification of different types of cell death, such as early apoptosis, late apoptosis and necrosis. For the assay, 1 x 10^5 cells were seeded into 12-well plate and 24 h later transfected with either vector control (VC) or optCPE expressing vector. At different time points cell culture supernatants were removed, cells were washed and centrifuged, followed by incubation with Annexin-V-FITC and PI-PE according to manufacturer's specification. For differentiation and quantification of vital, early-apoptotic and necrotic cells, FACS analysis was performed in VC and optCPE transfected cells. The Annexin-V and PI positive cells were quantified using the FACScalibur (Becton Dickinson) and data were expressed as percentage Annexin-V-FITC and PI-PE positive cells.

6. Caspase Assay

Caspase-Glo-3/7, -8 and -9 assays (Promega) were used to investigate the caspase-dependent cell death mechanism of CPE-treated tumor cells. For this, 1 x 10^5 cells were seeded into 12-well plate and 24 h later transfected with either vector control (VC) or optCPE plasmid DNA. At different time points supernatants were removed, cells were washed and incubated with Caspase-Glo reagent as recommended by manufacturer. After incubation luminescence was measured at 450 nm using a plate-reader luminometer (Tecan). Caspase activity was expressed as measured luminescence n-fold to VC.

7. Calpain-1/2 Assay

Calpain-1/2 activity was analyzed in optCPE-treated cells to determine the CPE-mediated cell death mechanism. For this, 1 x 10^5 cells were seeded into 12-well plate and after 24 h transfected with either VC or optCPE plasmid DNA. At different time points (6–72 h after transfection) cell culture supernatants were removed, cells were washed and Calpain-Glo Protease Assay (Promega) was applied as specified by manufacturer. The incubation with pro-luminescent substrate and reagent results in calpain-1/2 cleavage of substrate and luminescence signal, which was measured at 450 nm using a plate-reader luminometer (Tecan). The measured Calpain-Glo Assay signal was proportional to calpain-1/2 activity. Data was expressed as activated calpain-1/2 relative to µg protein

8. Lactate Dehydrogenase (LDH) Release Assay

The colorimetric Thermo ScientificTM PierceTM LDH Cytotoxicity Assay Kit was used to determine released LDH of optCPE-transfected cells to the medium. For this, 1 x 10^5 cells were seeded into 12-well plate and after 24 h transfected with either vector control (VC) or optCPE expressing vector. At different time points cell culture supernatants were collected and incubated with LDH reaction mixture according to manufacturer's recommendation. After incubation stop solution was added and the colorimetric LDH absorbance was measured at 490 nm and 680 nm using plate-reader (Tecan). LDH activity was determined by subtracting 680 nm absorbance from 490 nm absorbance values. Data was expressed as n-fold LDH release compared to VC.

9. IncuCyte Real-Time Live-Cell Analysis

To analyze CPE-mediated cell death in real time, the IncuCyte Live cell Analysis System (Essen BioScience) was used, which captured and analyzed images of optCPE treated cells over time. Here, different approaches were done to analyze optCPE mediated cytotoxicity using IncuCyteCytoRed reagent, detection of Ca2+ influx using calcium indicator Fluo-4 (Thermo Fisher Scientific), Caspase-3/7 activity using IncuCyte Caspase-3/7 reagent (Essen BioScience) or exposed PS in apoptotic/necrotic cells using IncuCyte Annexin-V reagent (Essen BioScience). For analyses, 1 x 10^5 cells were seeded into 12-well plate and 24 h later transfected with either VC or optCPE plasmid DNA, respectively. 6 h post-transfection, cells were treated with specific reagent according to manufacture's recommendation and plates were transferred to the IncuCyte. Images were taken automatically at defined time points and analysis of images was performed with IncuCyte software.

10. Analysis of Expression Profiles of Apoptosis-Related Proteins

The Proteome Profiler TM Array Human Apoptosis Array kit (R&D Systems, Minneapolis, MN, USA) was used to analyze the expression profiles of apoptosis-related proteins during CPE-mediated cell death. This array detects the relative expression levels of 35 apoptosis-related proteins, which were spotted in duplicate onto nitrocellulose membranes. For the analysis, 3 x 10^6 cells were seeded into 10 cm dishes and 24 h later transfected with vector control (VC) or optCPE plasmid DNA. As soon first signs of cell death were observed, cells were rinsed with PBS and solubilized in lysis buffer, and incubated on ice for 30 min. Then, cells were centrifuged at 14,000× *g* for 5 min and sample protein concentrations were quantified using BCA Protein Assay Kit (Pierce). Cell lysates were diluted and incubated overnight with human apoptosis array as recommended by manufacturer. To remove unbound proteins array was washed and then incubated with cocktail of biotinylated detection antibodies, followed by Streptavidin-HRP incubation. After another washing step, chemiluminescent detection reagent was applied, to visualize each captured spot corresponding to the amount of bound protein.

11. In Vivo Bioluminescence Imaging

For non-invasive bioluminescence imaging mice were anesthetized twice a week with Isofluran (Baxter, San Juan, Puerto Rico) and received intraperitoneally 150 mg kg^{-1} D-luciferin (Biosynth, Staad, Switzerland) dissolved in PBS. Imaging was performed with the NightOWL LB 981 system (Berthold Technologies, Bad Wildbad, Germany) with exposure times of 1 s and 60 s. ImageJ software version 1.50i was used for quantification and color-coding of the signal intensity. Overlay pictures were created with Adobe Photoshop CS5.1 software

12. Hematoxylin & e osin (HE) Staining

To gain overview of histopathological features and structural changes in treated pancreas tumors, tissue sections were stained with HE: tissues were fixed with isopropanol for 30 s and incubated with hemalum (Roth) for 2 min, resulting in blue stained nuclei,

followed by washing step with running tap water for 5 min and counterstaining with eosin (Roth) for 1 min. Then, slides were dehydrated through 95 % ethanol incubations (twice, 5 min each), 2 soaks in xylene (Sigma-Aldrich) for 5 min each and then covered with mounting medium. Tissue was evaluated in light microscope (Zeiss).

13. Ki67 Immunohistochemical Staining

Ki67 activity is a specific marker for cell proliferation and viability and was used to analyze whether optCPE gene transfer affects proliferation. For this, tissue sections were fixed with 4 % PFA for 10 min at RT and washed with PBS. The endogenous peroxidase was blocked with 3 % H_2O_2 for 5 min at RT, followed by another washing step. Sections were blocked with 20 % goat serum for 45 min and incubated with the primary antibody mouse anti-Ki67/MIB1 (hu) (Dianova, diluted 1:100 in AB-Diluent, Dako) in humidified chamber for 60 min at RT. After sections were washed, secondary antibody HRP-conjugated anti-mouse IgG (Jackson, diluted 1:400 in PBS) was added for 30 min at RT in humidified chamber and washed again in PBS. Then sections were incubated with DAB-chromogen substrate (DAKO) for 1–5 min, until appearance of brown staining, washed with ddH_2O and counterstained with hemalum, washed with running tap water for 5 min and covered with mounting medium (DAKO). Ki67 staining was captured with light microscope and blue and brown stained spots were counted with ImageJ software, the percentage of Ki67 was calculated and expressed as mean percentage of Ki67 positive cells.

14. Terminal Deoxynucleotidyl Transferase dUTP Nick End-Labeling (TUNEL) Assay

Appearance of DNA fragments as characteristic hallmark of apoptosis was visualized by using TumorTACS™ In Situ Apoptosis Detection Kit (Trevigen). The assay was performed as recommended by manufacturer. TUNEL staining was captured with light microscope and green and brown stained events were counted with ImageJ software, the percentage of TUNEL was calculated and expressed as mean counted value of TUNEL positive cells.

15. Determination of Vital vs. Necrotic Tumor Areas

To determine optCPE mediated intratumoral necrosis, shock frozen tumor tissues were fixed with Tissue-Tek Medium (Satura Tek) and dissected with the cryostat into cyro-sections each of 5 μm thickness and transferred onto cover slides. Sections were fixed with isopropanol for 30 s and incubated with hemalum (Roth) for 2 min, followed by washing step with running tap water for 5 min and counterstaining with eosin (Roth) for 1 min. Then, slides were dehydrated through 95 % ethanol incubations (twice, 5 min each), 2 soaks in xylene (Sigma-Aldrich) for 5 min each and then covered with mounting medium. Tissues were evaluated in light microscope (Zeiss) and images were analyzed for determination of vital vs. necrotic areas by using ImageJ software. Vital and necrotic areas are expressed as percentage of total tumor area in a given image.
